# Exposure to a Multitude of Environmental Chemicals During Pregnancy and Its Association with the Risk of Gestational Diabetes Mellitus

**DOI:** 10.3390/toxics13060461

**Published:** 2025-05-30

**Authors:** Yuzhe Lin, Xiong-Fei Pan, Maohua Miao, Huicai Guo, Peipei Meng, Wei Huang

**Affiliations:** 1 College of Environment and Climate, Guangdong Key Laboratory of Environmental Pollution and Health, Jinan University, Guangzhou 510632, China; lyz10@stu2022.jnu.edu.cn; 2 Section of Epidemiology and Population Health, Ministry of Education, Key Laboratory of Birth Defects and Related Diseases of Women and Children, West China Second University Hospital, Sichuan University, Chengdu 610041, China; pxiongfei@scu.edu.cn; 3 Shanghai-MOST Key Laboratory of Health and Disease Genomics, NHC Key Lab of Reproduction Regulation, Shanghai Institute for Biomedical and Pharmaceutical Technologies, Shanghai 200237, China; miaomaohua@163.com; 4 Department of Toxicology, School of Public Health, Hebei Medical University, Shijiazhuang 050017, China; huicaiguo@hebmu.edu.cn

**Keywords:** urine, ExpoNano, environmental chemicals, pregnant women, gestational diabetes mellitus

## Abstract

Gestational exposure to environmental chemicals has long been considered an important contributor to adverse pregnancy outcomes. While humans are exposed to a large complexity of environmental chemicals under real scenarios, existing studies have generally focused on a limited number of substances when exploring the health impacts of environmental exposure. Our work employed the recently developed ExpoNano strategy to characterize exposure to 283 environmental chemicals via urine in pregnant women from three Chinese cities and explored the association between environmental exposure and the risk of gestational diabetes mellitus (GDM) through a nested case–control study within a prospective birth cohort. The results revealed ubiquitous gestational exposure (detection frequency > 70%) to 37 chemicals, including selected mono-phthalate esters (mono-PAEs), non-PAE plasticizers, synthetic antioxidants, organophosphate esters, personal care products, UV stabilizers, photoinitiators, pesticides, and hydroxy polyaromatic hydrocarbons across the three cities. The cumulative concentrations of detectable chemicals displayed median values of 461–741 ng/mL in different populations of pregnant women, which exhibited significant variations across regions. In the GDM case–control study (85 GDM cases and 170 healthy controls), although mixed exposure was not associated with the risk of GDM, exposure to acetyl tributyl citrate, an emerging plasticizer, was found to be significantly associated with GDM risk, based on both the single-pollutant and mixed exposure models. However, it should be noted that due to the relatively small sample size, the findings should be interpreted as preliminary exploratory results requiring further validation in larger cohorts. This study demonstrates the complexity of environmental chemical exposure during pregnancy, indicating a critical need for further investigations of the potential impact on pregnancy outcomes.

## 1. Introduction

Gestational exposure to environmental chemicals has been considered an important risk factor inducing adverse effects on pregnancy outcomes, such as gestational diabetes mellitus (GDM) and unfavorable birth outcomes [1,2,3,4,5]. Available studies have reported a diversity of environmental chemicals commonly found in pregnant women, including, but not limited to, flame retardants, per- and polyfluoroalkyl substances (PFAS), personal care products (PCPs), and many others [6,7,8,9,10]. These exogenous chemicals vary largely in regards to chemical properties and biological effects, and the mixture of them may produce cocktail effects different from what can be predicted from individual compounds. Although epidemiological investigations have identified associations between gestational exposure to discrete chemical classes, including organophosphate esters (OPEs), polycyclic aromatic hydrocarbons (PAHs), and GDM risk [11,12], and experimental toxicological studies have further substantiated the pathophysiological mechanisms linking specific OPEs and PAHs to metabolic dysregulation [13,14], a critical methodological constraint persists. Current chemical-centric approaches fail to adequately address the exposome-level complexity inherent to real-world scenarios, where pregnant women experience continuous, multi-modal exposure to complex mixtures comprising hundreds of structurally and toxicologically diverse environmental contaminants [15]. This paradigm gap fundamentally limits our capacity to discern mixture-driven etiological links between cumulative chemical exposures and pregnancy complications like GDM. Therefore, comprehensive and high-throughput characterization of gestational exposure profiles constitute a solid foundation for exploring potential effects on pregnancy outcomes.

Urine analysis has been demonstrated as an efficient biomonitoring approach to capturing a large variety of endogenous chemicals or their metabolites [16,17,18,19]. It also exhibits a unique advantage over the collection of other biological fluids as a noninvasive and convenient sampling method for human biomonitoring. However, traditional urine analysis via liquid–liquid extraction (LLE) or solid–liquid extraction (SLE) faces challenges to meet high-throughput determination of various environmental chemicals, due to the high dependency on solvent or sorbent options. Most studies generally employed LLE or SLE methods to analyze urinary residues of a specific group of compounds [20]. Recently, Song et al. developed an ExpoNano strategy to achieve urinary exposome assessment of human exposure to environmental chemicals [21]. ExpoNano is based on the ultra-highly efficient trapping of organic molecules from urine by our fabricated hyper-crosslinked polymers (HCPs) due to their ultrahigh Brunauer–Emmett–Teller (BET) specific surface areas, high intrinsic porosities, and good thermal and chemical stabilities. Solid validation tests have demonstrated that the ExpoNano strategy can capture organic molecules with a wide range of molecular weights (75 to 837 Da) and log Kow values (−9.86 to 10.56). Therefore, our ExpoNano strategy offers a novel and ultrahigh-throughput measurement of environmental chemicals in urine.

In this study, we aimed to employ ExpoNano to characterize the exposure profiles of Chinese pregnant women to a multitude of environmental chemicals in populations from different regions. This could facilitate the understanding of common and region-specific exposure profiles in pregnant women. Additionally, we explored the association between chemical exposure and the risk of GDM based on a small-scale nested case–control study established based on one of the study populations. This pilot investigation aimed to identify environmental chemicals with high GDM risks, constituting a foundation for further validation with larger-scale studies and the exploration of potential mechanisms specific to the health impacts.

## 2. Materials and Methods

### 2.1. Study Populations and Sample Collection

Our study populations consisted of pregnant women recruited from three Chinese cities (i.e., Shanghai, Shijiazhuang, and Chengdu) located in East, North, and West China, respectively. The participants were recruited through the Maternal and Child Healthcare Hospital of Minhang District, Shanghai (n = 97); the Fourth Hospital of Hebei Medical University (n = 135), and the Maternal and Child Health Hospital of Shuangliu District, Chengdu (n = 255) (Table S1). Spot urine was collected from each participant during the first or second trimester and stored at −80 °C prior to sample analysis. The samples from the Chengdu population also constituted a prospective nested GDM case–control study which included 85 cases and 170 matched healthy controls. In the case-control study, all covariates (e.g., baseline BMI, lifestyle factors) and urine samples for chemical analysis were collected in the first trimester (<16 week) prior to GDM diagnosis (24-28 week), which isolates exposure effects from post-diagnosis behavioral adaptations (Table S2). Each case was randomly matched with two controls based on age (±3 years) and gestational age (±4 weeks). The participants provided written informed consent prior to participation in the study. Most participants (except for the Shijiazhuang population) were interviewed shortly after enrollment by trained investigators or nurses to complete structured questionnaires on maternal sociodemographic characteristics, lifestyle habits, and disease history. The study was approved by the Ethics Committee of Jinan University; the Ethics Committee of Tongji Medical College, Huazhong University of Science and Technology; and the Ethics Committee of Shanghai Institute for Biomedical and Pharmaceutical Technologies (formerly Shanghai Institute of Planned Parenthood Research).

### 2.2. Sample Analysis

Pre-treatment of urine samples with the ExpoNano strategy followed the approach established in a previous study [21]. In brief, after an aliquot of 0.5 mL of thawed urine was spiked with surrogate standards, 100 µL of 1 M ammonium acetate and 20 µL of β-glucuronide polymerase (2500 units/mL) were added sequentially to the mixture, which was then vortexed and placed in a 37 °C water bath and incubated at a constant temperature overnight (for at least 6 h). Following enzymatic hydrolysis, the mixture was spiked with 5 mg of HCP particles and oscillated for 5 min. After centrifugation at a speed of 13,000 rpm and removal of the supernatant, the collected HCP particles were immersed in 1.5 mL of methanol (for 5 min) for chemical extraction. The extraction was repeated once with 1.5 mL of ethyl acetate. The combined extract was concentrated to 100 µL and spiked with internal standards prior to instrumental analysis.

Quantitative determination of urinary residues was achieved on a Shimadzu ultraperformance liquid chromatograph coupled to a 5500 triple quadrupole mass spectrometer (AB Sciex, Toronto, ON, Canada). The target compounds include nine groups of environmental chemicals with available reports showing their existence in human urine. They include monophthalate esters (mono-PAEs), non-PAE plasticizers (NPPs), personal care products (PCPs), synthetic antioxidants (SAOs), organophosphate esters (OPEs), UV stabilizers (UVs), hydroxy polycyclic aromatic hydrocarbons (OH-PAHs), photoinitiators (PIs), pesticides (PTs), brominated flame retardants (BFRs), and retarders (RTs), totaling 283 target chemicals for our analysis (Table S3). The details of the instrumental analysis, as well as the parameters, are summarized in Table S4.

### 2.3. Quality Assurance and Control

The performance of the ExpoNano-based strategy, including the extraction efficiency and matrix effect of target compounds, has been determined through matrix spiking tests, and the results, with details, have been reported in a previous work [20]. The analysis revealed that the target compounds displayed an average recovery efficiency of 60 ± 10% to 135 ± 6%, and their matrix effects ranged from 73.4 ± 10% to 137 ± 20%. A laboratory blank (i.e., HPLC water) was prepared for every ten samples to assess the laboratory environment and possible contamination caused by the pre-treatment process. Fourteen compounds were detected in the blank at concentrations ranging from 0.01 to 0.31 ng/mL (Table S4), and the blanks were subtracted from the final results of the samples.

### 2.4. Data Analysis

The limit of quantification (LOQ) of an analyte was defined as a concentration corresponding to an instrumental response 10 times the standard deviation of the noise when the analyte was not detectable in the procedural blanks. For compounds with blank contamination, the LOQ was defined as the average blank contamination plus 10 times the standard deviation of the blank contamination determined in the procedural blanks. The LOQ of the compounds ranged from 0.0015 to 0.575 ng/mL (Table S5). Concentration data (ng/mL) were reported after correction for the recoveries of corresponding surrogate standards and urine specific gravity. Analytes with a detection rate higher than 70% were included in subsequent data analysis. Any measurements below the LOQ were replaced with LOQ/√2 for statistical analysis.

The Mann–Whitney U test was used to analyze the significance of differences between medians of independent samples (GDM cases versus healthy controls). Binary logistic regression models were used to estimate odds ratios (ORs) and 95% confidence intervals (CIs) to determine the association between pollutant exposure and GDM risk, after adjustment with the covariates, including maternal age (continuous), pre-pregnancy BMI (continuous), maternal education (<associate degree or> associate degree), alcohol drinking (yes or no), smoking during pregnancy (yes or no), and parental history of diabetes (yes or no). To account for multiple testing events, two-sided *p* values were adjusted according to the method of Benjamini/Hochberg (B/H) to control the false discovery rate (FDR) [22]. An association was considered to be statistically significant if its corresponding B/H-adjusted *p* value was below 0.05, corresponding to an FDR of 5%.

The relationship between combined exposure to the pollutant mixture and GDM risk was analyzed by Bayesian kernel machine regression (BKMR) models. The joint effect was determined by estimating the differences in GDM risk when all of the chemicals were held at their 25th to 75th percentile levels (in five percentile point increments) as compared to when they were fixed at their 50th percentile rate. The single-chemical association was identified by estimating the change in GDM risk associated with a change in a single chemical is at the 75th vs 25th percentile, while all the other chemicals are fixed at either the 25th, 50th, or 75th percentile. We fitted all BKMR models with binomial distributions using 100 knots and 50,000 Markov chain Monte Carlo iterations. The posterior inclusion probability (PIP) for each predictor variable was calculated as a measure of variable importance. A PIP threshold of 0.5 was used to determine the relative importance of each chemical to the risk of GDM.

All statistical analyses were performed using SPSS (IBM SPSS Statistics 26.0) and R (version 4.2.2; R Development Core Team). The statistical significance of all statistical analyses was set at α = 0.05.

## 3. Results

### 3.1. Characterization of Exposure Profiles

**Figure 1:**
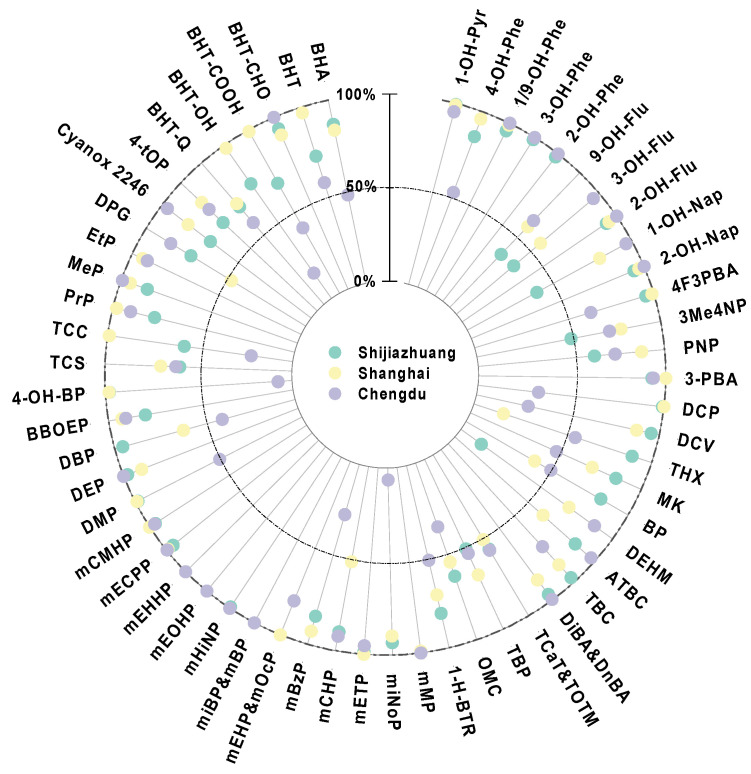
Detection frequency of target analytes in the urine of pregnant women from three cities. Only the compounds with an overall detection frequency > 50% in all subjects are included. Point overlap exists if the compounds display the same detection rate, e.g., mEHHP has a detection frequency of 100% in all three cities.

Among the target environmental chemicals, a total of 80 compounds were detected in the urine of pregnant women from different regions. A total of 37 these exhibited a detection frequency > 70%, indicating a complex exposure scenario across regions (Figure 1). Mono-PAEs appeared to be the most abundant group of chemicals, with the median concentrations of 373–618 ng/mL across regions, followed by SAOs (19.6–48.0 ng/mL), NPPs (11.0–57.2 ng/mL), PCPs (7.49–53.3 ng/mL), OH-PAHs (4.54–42.8 ng/mL), OPEs (14.3–23.5 ng/mL), PTs (9.29–11.2 ng/mL), UVs (1.31–4.26 ng/mL), and PIs (0.02–1.88 ng/mL). The total concentrations of all detected compounds displayed a median value of 461–741 ng/mL across the regions (Table S5).

The data revealed ubiquitous exposure to a complexity of environmental chemicals. Other than well-known chemicals such as mono-PAEs, OH-PAHs, PCPs, and pesticides, a few emerging contaminants also exhibited ubiquitous exposure in pregnant women. For example, along with the restrictions of phthalate ester plasticizers in consumer products, alternative chemicals have been increasingly used as plasticizers or as PAE replacements [23,24]. Indeed, our study found a more than 80% detection rate for di-n-butyl/diisobutyl adipate (DnBA/DiBA, co-elution, 5.93–38.2 ng/mL), tributyl citrate (TBC, 0.85–4.06 ng/mL), and acetyl tributyl citrate (ATBC, 1.70–16.0 ng/mL) in urine, indicating widespread exposure in pregnant women. These are chemicals representative of adipates and citric acid esters. TBC and ATBC have been used as plasticizers in food contact polymer materials, while DnBA/DiBA is widely used as a plasticizer in personal care products [25,26,27,28]. Although the levels of NPPs are generally one order of magnitude lower than that of PAEs, the restrictions of PAEs may result in increasing applications of many NPPs and subsequently, in incrementally increased human exposure. While several NPPs (e.g., DnBA/DiBA and ATBC) are listed as high production volume (HPV) chemicals, knowledge regarding their exposure levels in human remains very limited.

Other chemicals of emerging concern which are commonly found in urine included several UV stabilizers and antioxidants. 2,6-di-tert-butyl-4-hydroxytoluene (BHT) and its derivatives constituted a major portion of synthetic antioxidants in urine. BHT has been broadly applied as an antioxidant to cosmetics, rubbers, plastics, and even food [29,30], subsequently leading to widespread human exposure. Although most derivatives were suggested to be less toxic than BHT, BHT-quinol (BHT-Q) has been reported to exhibit greater toxicity than does BHT [31]. The ubiquitous detection of BHT and its derivatives raises concern regarding their potential impact on pregnancy health. Other than BHT and its derivatives, 1,3-diphenylguaidine (DPG) was also frequently detected in pregnant women. DPG is applied as an antioxidant to polymers and rubber products [32,33,34], but to date, very few studies have reported human exposure to this emerging chemical. 

**Figure 2:**
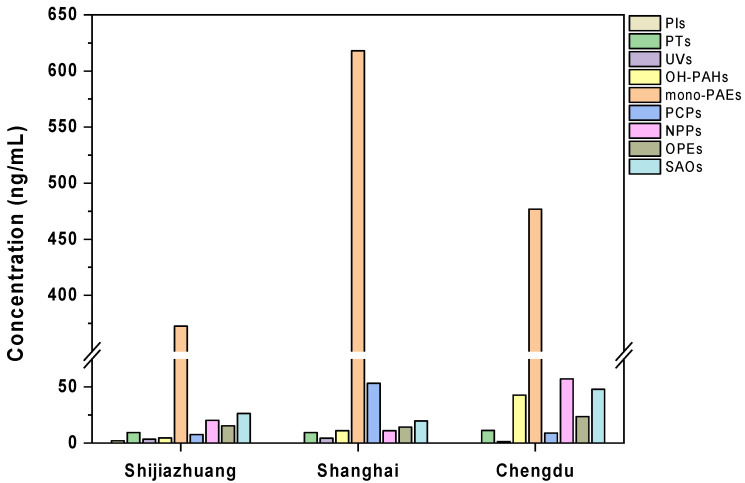
Multiple bar chart showing the concentrations of nine groups of exogenous chemicals in the urine of pregnant women in each city. Mono-PAEs, monophthalate esters; SAOs, synthetic antioxidants; OPEs, organophosphate esters; NPPs, non-PAE plasticizers; PCPs, personal care products; OH-PAHs, hydroxy polycyclic aromatic hydrocarbons; UVs, UV stabilizers; PTs, pesticides; PIs, photoinitiators.

Urinary concentrations of environmental chemicals exhibited significant variations in pregnant women from different regions (*p* = 0.004, F_2,484_ = 5.55, ANOVA) (Table S6). The highest concentrations of all detectable chemicals combined were found in pregnant women from Shanghai, whereas the lowest were measured in the population from Shi Jiazhuang (Figure 2). The same pattern was also observed for most individual groups of chemicals, although the composition profiles of each group of chemicals did not vary largely across populations (Figure S1). Such a pattern may reflect regional variation in economic development, since the city of Shanghai has the highest gross domestic production (GDP) among all three regions. However, the differences between regions may also be confounded by the variations in demographic characteristics (e.g., age and body mass index), as well as the inconsistency of the sampling stage (i.e., early or mid-term pregnancy) across populations. As our study did not collect sufficient data regarding sociodemographic information for some study populations, we will explore the factors causing regional exposure differences in future investigations.

### 3.2. Association Between Chemicals Exposure and GDM

We established a small-scale prospective nested case–control study (85 GDM cases and 170 healthy controls) based on the Chengdu population to explore whether the urinary concentration of environmental chemicals is linked with GDM risk. Chemicals with a detection rate higher than 70% in urine samples were included in the analysis. Among them, ATBC exhibited significantly higher concentrations in the case group than in the control group (Table 1).

**Figure 3:**
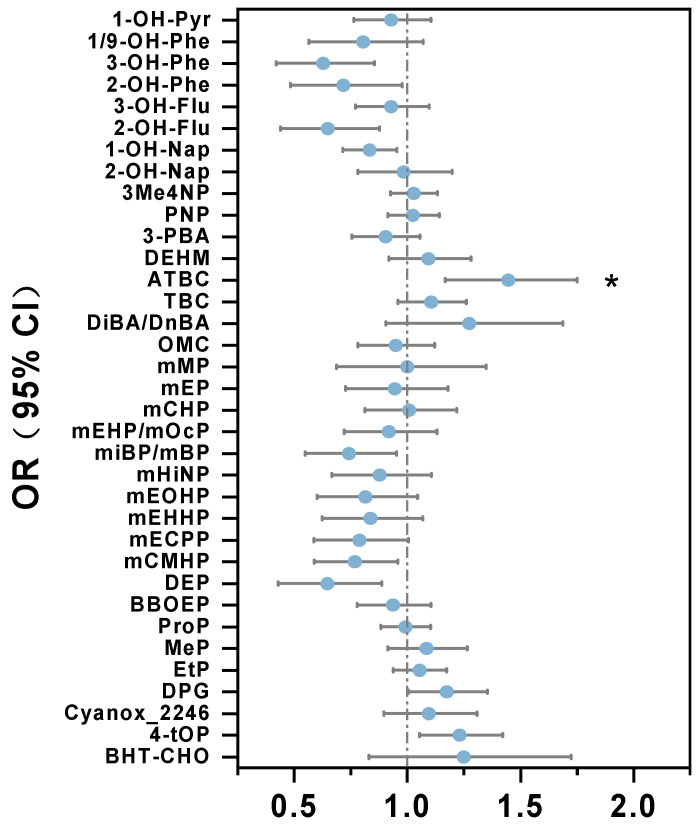
Association of concentrations of highly urinary-detectable compounds (DF > 70%, SG-corrected) with gestational diabetes mellitus in pregnant women in Chengdu, China. The model was adjusted for maternal age, pre-pregnancy BMI, income, maternal education, parental history of diabetes, smoking, and alcohol drinking. To control for the false discovery rate (FDR), we adjusted the bilateral *p*-values according to the Benjamini/Hochberg (B/H) method. * BH-adjusted *p* value < 0.05.

Further logistic regression analysis explored the association between early gestational exposure to environmental chemicals and the GDM risk, after adjustment with potential covariates. The results revealed a significant and positive association between urinary levels of ATBC and GDM risk (OR = 1.46; 95% CI: 1.03, 2.08), (Figure 3, Table S7). Some other substances showed a positive, but not statistically significant, association with GDM.

Toxicological investigations are still lacking or insufficient for most commonly detected compounds, especially for the identified key compounds. The lack of toxicological threshold data largely limits our ability to quantitatively assess the risk of human exposure to specific endpoints. In addition, the presence of different classes of compounds in pregnant women raises concerns about the health effects of exposure to this cocktail of environmental chemicals. We further used the BKMR model to eliminate collinearity between environmental chemicals with a DF > 70% and to assess their joint effects. The results showed a positive, but not significant, association between mixed exposure and the risk of GDM (Figure S3a). However, when examining the individual effects of each compound within the mixture, a significant and positive correlation with the risk of GDM was found as the exposure to ATBC increased from the 25th to the 75th percentile when the other compounds were fixed at a particular threshold (25th, 50th, or 75th percentile) (Figure S3b). In addition, the highest PIP value was observed for ATBC (Table S8).

In summary, ATBC could play an important role in the link between environmental exposure and the risk of GDM in pregnant women. Gestational exposure to ATBC at environmentally relevant concentrations has been reported to disrupt glucose metabolism homeostasis in female C57BL/6J mice [35]. This exposure could elevate liver lipid levels and insulin resistance in F1 female mice and increase weight gain, liver triglycerides, and fasting blood glucose levels in F2 male mice, indicating intergenerational disturbance of glucose metabolism across generations of mice [35]. Further in vivo and in vitro studies also revealed obesogenic and fatty acid-inducing effects of ATBC at sub-chronic exposure levels [36]. Consistent with these in vivo and in vitro studies, our work also suggested that early gestational ATBC exposure could interfere with glucolipid metabolism in pregnant women.

### 3.3. Implications

Our study revealed ubiquitous exposure to a complexity of environmental chemicals in pregnant women from different regions. The case–control study further reported a significant association between ATBC exposure during early pregnancy and the risk of GDM. Although our study was limited by a relatively small sample size and variations in sociodemographic characteristics of the populations across regions, our study has several important implications.

An increasing number of emerging contaminants complicates gestational exposure scenarios. Despite the fact that our work has included more than 280 environmental chemicals, many emerging chemicals have been subjected to large-scale applications but have received little attention. In particular, various chemicals have been produced to replace traditional chemicals that have been completely phased out or which are subject to restrictions in certain applications. This increases the complexity of human exposure and the subsequent biomonitoring or health assessments. As our ExpoNano strategy can achieve ultra-highly efficient trapping of organic molecules with a wide range of molecular weights and log Kow values, it can be used to expand our screening scope on the basis of more comprehensive chemical databases.

Urinary biomonitoring requires better identification of exposure markers of environmental chemicals. Our current screening list includes mainly parent compounds. However, many exogenous chemicals may be subjected to phase I and/or phase II transformation, which produces a series of metabolites. Thus, the analysis of parent compounds may not be appropriate for urinary biomonitoring. Identification of the most important metabolites as exposure markers becomes critical to precisely reflect human exposure. Spot urine measurements may be influenced by physiological variability (e.g., hydration status, timing) and are less precise than 24-h urine collection for certain biomarkers; however, the latter is often impractical in large-scale epidemiological studies due to high participant burden, logistical complexity, and risks of incomplete sampling. In addition, while urine sampling is widely adopted in epidemiological studies due to its non-invasive nature and logistical feasibility, we acknowledge its limitations for assessing certain environmental toxicants (e.g., lead, methyl mercury, etc.). Future work should integrate multi-matrix approaches (e.g., paired blood–urine sampling) to refine the exposure assessments for specific toxicants.

The impact of the complexity of environmental exposure on pregnancy outcomes requires better investigations. Most available studies generally focus on a limited number or group of chemicals when exploring environmental impact on adverse pregnancy outcomes. This overlooks the complexity of environmental exposure, which may produce cocktail effects different from what can be predicted from single compound exposure. Our study provided a small-scale investigation of the potential impact of mixed exposure on GDM. However, although the GDM study was based on a prospective cohort, subclinical metabolic shifts during the prediabetic phase might influence both exposures and outcomes, thereby leading to reverse causality. Future studies should employ Mendelian randomization or longitudinal behavioral monitoring to disentangle these temporal relationships. Larger-scale studies are needed to better identify the most important exposure markers contributing to health outcomes via mixed exposure models and validation by animal studies.

## 4. Conclusions

Our study employed the ExpoNano strategy to characterize gestational exposure to a large complexity of environmental chemicals in pregnant women from three different regions across China. The results revealed ubiquitous exposure of pregnant women to diverse chemicals, with significant variations in combined exposure levels across regions. Based on a nested case–control study, consistent associations between ATBC exposure and GDM risk in both single-pollutant and mixture exposure models were found, highlighting this plasticizer substitute as a priority chemical for pregnancy health surveillance. Nevertheless, our findings may be limited by the small sample size and heterogeneity of the study population. Validation of these findings requires larger-scale cohort studies and evidence from animal exposure studies. Collectively, our findings demonstrate that pregnant women are exposed to a complexity of environmental chemicals with diverse applications and physiological properties. Further research is critically needed to better investigate the impact of co-exposure to various chemicals on pregnancy outcomes, to identify the key compounds exerting the greatest contribution to adverse health effects, and to elucidate the underlying molecular mechanisms of these chemicals.

## Figures and Tables

**Table 1:** Concentration and detection frequency (DF, %) of chemicals in the urine of pregnant women in the GDM case and control groups (ng/ml).

Chemicals	non-GDM (n = 170)		GDM (n = 85)		FDR
	Median (ng/mL)	DF (%)	Median (ng/mL)	DF (%)	
BHT-CHO	30.5	100%	32.4	100%	0.507
4-tOP	6.04	75%	9.75	88%	0.090
Cyanox_2246	0.350	98%	0.420	94%	0.261
DPG	1.98	82%	3.04	89%	0.343
EtP	0.972	90%	1.11	93%	0.968
MePa	6.50	99%	6.74	100%	0.513
ProP	0.662	89%	0.722	93%	0.625
BBOEP	1.44	91%	1.60	91%	0.872
DEP	24.6	100%	16.2	100%	0.000
mCMHP	5.70	97%	4.26	96%	0.064
mECPP	6.02	100%	4.93	99%	0.145
mEHHP	14.7	100%	11.2	100%	0.175
mEOHP	4.33	100%	3.02	100%	0.145
mHiNP	7.86	100%	5.29	100%	0.208
miBP_mBP	62.5	100%	44.8	100%	0.145
mEHP_mOcP	0.865	79%	0.712	82%	0.508
mCHP	1.77	91%	1.94	94%	0.669
mEP	3.10	97%	2.22	92%	0.548
mMP	0390	100%	0.369	100%	0.815
OMC	1.19	86%	1.69	88%	0.160
DiBA_DnBA	35.5	100%	46.7	99%	0.330
TBC	0.712	74%	1.12	78%	0.064
ATBC	13.4	96%	24.3	99%	0.018
DEHM	0.444	88%	0.779	88%	0.145
3-PBA	2.04	95%	2.27	89%	0.968
PNP	3.25	72%	4.40	75%	0.754
3Me4NP	3.16	71%	3.07	74%	0.880
2-OH-Nap	16.1	100%	14.6	100%	0.855
1-OH-Nap	11.8	98%	9.01	93%	0.145
2-OH-Flu	7.46	100%	4.87	100%	0.061
3-OH-Flu	0.445	96%	0.413	94%	0.588
2-OH-Phe	2.50	100%	2.04	100%	0.145
3-OH-Phe	4.27	100%	3.25	100%	0.058
1/9-OH-Phe	2.64	100%	1.98	100%	0.282
1-OH-Pyr	0.193	97%	0.157	92%	0.855

## Data Availability

The raw data supporting the conclusions of this article will be made available by the authors on request.

## Supplementary Materials

The following supporting information can be downloaded at: https://www.mdpi.com/article/10.3390/toxics13060461/s1, Table S1: Age and prenatal BMI of pregnant women in three cities. Table S2: Characteristics of the study population in Chengdu (n = 255). Table S3: Summary of Target Exogenous Chemicals. Table S4: Major chemical-dependent parameters for instrumental analysis. Table S5: Detection frequency (DF, %) and median concentrations (ng/mL) of exogenous chemicals in the urine of pregnant women in three cities. Table S6: Results of one-way ANOVA analysis. Table S7: Association between concentrations of highly detected chemicals in the urine of pregnant women and the risk of gestational diabetes mellitus. Table S8: Posterior inclusion probabilities (PIPs) from BKMR. Figure S1: Composition profiles of major compounds in the urine of pregnant women in three cities. Figure S2: Differences in the concentration of chemicals (DF > 70%) in the urine of pregnant women in the case and control groups. Figure S3: Association of mixed chemicals exposure with GDM assessed by BKMR.
