# Supplementary material for: Exposure to a Multitude of Environmental Chemicals During Pregnancy and Its Association with the Risk of Gestational Diabetes Mellitus
Source: Toxics. 2025 May 30;13(6):461. doi: 10.3390/toxics13060461 (PMC12197530; doi:10.3390/toxics13060461)
Supplement: Supplementary file 1 [file toxics-13-00461-s001.zip › toxics-3621971-supplementary.pdf]

Supplementary Appendix

Exposure to a Multitude of Environmental Chemicals During Pregnancy and its Association with the Risk of Gestational Diabetes Mellitus

Table S1 Age and prenatal BMI of pregnant women in three cities.

Table S2. Characteristics of the study population in Chengdu (n = 255).

Table S3. Summary of target exogenous chemicals.

Table S4. Major chemical-dependent parameters for instrumental analysis.

Table S5. Detection frequency (DF, %) and median concentrations (ng/mL) of exogenous chemicals in the urine of pregnant women in three cities.

Table S6. Results of one-way ANOVA analysis.

Table S7. Association between concentrations of highly detected chemicals in the urine of pregnant women and the risk of gestational diabetes mellitus.

Table S8. Posterior inclusion probabilities (PIPs) from BKMR.

Figure S1. Composition profiles of major compounds in the urine of pregnant women in three cities.

Figure S2. Differences in the concentration of chemicals (DF>70%) in the urine of pregnant women in the case and control groups.

Figure S3. Association of mixed chemicals exposure with GDM, assessed by BKMR.

Table S1. Age and prenatal BMI of pregnant women in three cities.

	Shi Jiazhuang	Shang Hai	Cheng Du
Maternal age (years)	30.6±4.7	29.2±3.0	**27.5±4.0**
Pre-pregnancy BMI[a] (kg/m^2)	/	21.0±2.5	21.5±3.0

Notes: /: Data on prenatal BMI was not available at the time of collection of this sample.

Table S2. Characteristics of the study population in Chengdu (n = 255).

Variable	Total Population (n=255)	GDM (n=85)	Non-GDM (n=170)
Maternal age (years)	27.5±4.0	27.5±3.8	27.5±4.3
Delivery week	38.8±1.3	38.7±1.3	38.9±1.2
Pre-pregnancy BMI[a] (kg/m^2)	21.5±3.0	22.3±2.8	21.1±3.1
Income (CNY[b]/year)			
<30,000	26 (10.2%)	5 (5.9%)	21 (12.4%)
30,000-70,000	68 (26.7%)	18 (21.2%)	50 (29.4%)
>70,000	161 (63.1%)	62 (72.9%)	99 (58.2%)
Maternal education			
< Associate degree	143 (56.1%)	52 (61.2%)	91 (53.5%)
> Associate degree	112 (43.9%)	33 (38.8%)	79 (46.5%)
Parental history of diabetes	26 (10.2%)	16 (18.8%)	20 (11.8%)
Smoking	10 (3.9%)	4 (4.7%)	6 (3.5%)
Alcohol drinking	47 (18.4%)	20 (23.5%)	27 (15.9%)

Notes: [a] BMI: body mass index; [b] CNY: Chinese yuan.

Table S3. Summary of target exogenous chemicals. This table provides a summary of the target exogenous chemicals and the corresponding alternative standards used to develop the HCP analytical protocol, including their abbreviated or commercial names, full names, CAS numbers, reference standard sources, corresponding alternative standards for individual chemicals, and information on the reagents used in the study.

Abbreviation or commercial name	Full name	CAS number	Supplier[a]	Surrogate standards
		**Target exogenous chemicals**		
Organophosphate esters (OPEs)				
TEP*	triethyl phosphate	78-40-0	AccuStandard	TEP-d_{15}
TPrP	tripropyl phosphate	513-08-6	AccuStandard	TEP-d_{15}
TBP*	tributyl phosphate	126-73-8	AccuStandard	TBP-d_{27}
TPHP*	triphenyl phosphate	115-86-6	AccuStandard	TPHP-d_{15}
TCrP	tricresyl phosphate	1330-78-5	AccuStandard	TPHP-d_{15}
TEHP*	tris(2-ethylhexyl) phosphate	78-42-2	AccuStandard	M6TBOEP
TBOEP	tris(2-butoxyethyl) phosphate	78-51-3	AccuStandard	M6TBOEP
TCEP	tris(2-chloroethyl) phosphate	115-96-8	AccuStandard	TCEP-d_{12}
TCIPP	tris(2-chloroisopropyl) phosphate	13674-84-5	AccuStandard	TCEP-d_{12}
TDCIPP	tris(1,3-dichloro-2-propyl) phosphate	13674-87-8	AccuStandard	TDCIPP-d_{15}
TDBPP	tris(2,3-dibromopropyl) phosphate	126-72-7	AccuStandard	TDCIPP-d_{15}
T34DMPP	tris(3,4-dimethyphenyl) phosphate	62-11-1	Wellington	TPHP-d_{15}
T35DMPP	tris(3,5-dimethylphenyl) phosphate	25653-16-1	Wellington	TPHP-d_{15}

BPADP	bisphenol A bis(diphenyl phosphate)	5945-33-5	AccuStandard	TPHP-d_{15}
CDP	cresyl diphenyl phosphate	26444-49-5	AccuStandard	TPHP-d_{15}
EHDPHP	2-ethylhexyl-diphenyl phosphate	1241-94-7	AccuStandard	TPHP-d_{15}
IDDPP	isodecyl diphenyl phosphate	29761-21-5	AccuStandard	TPHP-d_{15}
RDP	resorcinol bis(diphenyl phosphate)	57583-54-7	AccuStandard	TPHP-d_{15}
V6	tetrakis(2-chloroethyl)dichloroisopentyl diphosphate	38051-10-4	AccuStandard	TPHP-d_{15}
2IPPDPP	2-isopropylphenyl diphenyl phosphate	64532-94-1	Wellington	TEP-d_{15}
4IPPDPP	4-isopropylphenyl diphenyl phosphate	55864-04-5	Wellington	TEP-d_{15}
24DIPPDPP	2,4-diisopropylphenyl diphenyl phosphate	96107-55-0	Wellington	TEP-d_{15}
B2IPPPP	bis(2-isopropylphenyl) phenyl phosphate	69500-29-4	Wellington	TEP-d_{15}
B4IPPPP	bis(4-isopropylphenyl) phenyl phosphate	55864-07-8	Wellington	TEP-d_{15}
B24DIPPPP	bis(2,4-diisopropylphenyl) phenyl phosphate	2190501-29-0	Wellington	TEP-d_{15}
T2IPPP	tris(2-isopropylphenyl) phosphate	64532-95-2	AccuStandard	TEP-d_{15}
T3IPPP	tris(3-isopropylphenyl) phosphate	72668-27-0	Wellington	TEP-d_{15}
T4IPPP	tris(4-isopropylphenyl) phosphate	2502-15-0	Wellington	TEP-d_{15}
2tBPDPP	2-tert-butylphenyl diphenyl phosphate	83242-23-3	Wellington	TBP-d_{27}
4tBPDPP	4-tert-butylphenyl diphenyl phosphate	981-40-8	Wellington	TBP-d_{27}
B2tBPPP	bis(2-tert-butylphenyl) phenyl phosphate	65652-41-7	Wellington	TBP-d_{27}
B4tBPPP	bis(4-tert-butylphenyl) phenyl phosphate	115-87-7	Wellington	TBP-d_{27}
T4tBPP	tris(4-tert-butylphenyl) phosphate	78-33-1	Wellington	TBP-d_{27}
DMP*	dimethyl phosphate	813-78-5	AccuStandard	TEP-d_{15}
DEP*	diethyl phosphate	598-02-7	AccuStandard	TEP-d_{15}
DBP*	dibutyl phosphate	107-66-4	Sigma-Aldrich	DBP-d_{18}
DPHP*	diphenyl phosphate	838-85-7	Sigma-Aldrich	TPHP-d_{15}
DoCP	di-o-tolyl phosphate	35787-74-7	TRC	DoCP-d_{14}
DpCP	di-p-tolyl-phosphate	843-24-3	TRC	DpCP-d_{14}

BEHP	bis(2-ethylhexyl) phosphate	298-07-7	Sigma-Aldrich	BEHP-d_{34}
BBOEP*	bis(butoxyethyl) phosphate	14260-97-0	TRC	BBOEP-d_8
BCEP	bis(2-chloroethyl) phosphate	3040-56-0	TRC	BCEP-d_8
BCIPP	bis-(1-chloro-2-propyl) phosphate	789440-10-4	TRC	BCIPP-d_{12}
BDCIPP	bis(1,3-dichloro-2-propyl) phosphate	72236-72-7	Wellington	BDCIPP-d_{10}
BDBPP	bis(2,3-dibromopropyl) phosphate	5412-25-9	AccuStandard	BDCIPP-d_{10}
Phenolic polycyclic aromatic hydrocarbons (OH-PAHs)				
1-OH-Nap*	1-hydroxynaphthalene	90-15-3	AccuStandard	2-OH-Nap-d_8
2-OH-Nap*	2-hydroxynaphthalene	135-19-3	Chiron AS	2-OH-Nap-d_8
2-OH-Flu*	2-hydroxyfluorene	2443-58-5	TRC	3-OH-Flu-d_9
3-OH-Flu*	3-hydroxyfluorene	6344-67-8	TRC	3-OH-Flu-d_9
9-OH-Flu*	9-hydroxyfluorene	1689-64-1	TRC	3-OH-Flu-d_9
3-OH-Fla	3-hydroxyfluoranthene	17798-09-3	TRC	3-OH-Flu-d_9
1-OH-Pyr*	1-hydroxypyrene	5315-79-7	AccuStandard	1-OH-Pyr-d_9
1-OH-Phe*	1-hydroxyphenanthrene	2433-56-9	Chiron AS	3-OH-Phe-$^{13}C_4$
2-OH-Phe*	2-hydroxyphenanthrene	605-55-0	Dr. Ehrenstorfer	3-OH-Phe-$^{13}C_4$
3-OH-Phe*	3-hydroxyphenanthrene	605-87-8	Dr. Ehrenstorfer	3-OH-Phe-$^{13}C_4$
4-OH-Phe*	4-hydroxyphenanthrene	7651-86-7	TRC	3-OH-Phe-$^{13}C_4$
9-OH-Phe*	9-hydroxyphenanthrene	484-17-3	TRC	3-OH-Phe-$^{13}C_4$
3-OH-BaP	3-hydroxybenzo[a]pyrene	13345-21-6	TRC	1-OH-Pyr-d_9
3-OH-BcP	3-hydroxybenzo[c]phenanthrene	22717-95-9	TRC	1-OH-Pyr-d_9
6-OH-Chr	6-hydroxychrysene	37515-51-8	TRC	1-OH-Pyr-d_9
Personal care products (PCPs)				
MeP*	methyl paraben	99-76-3	AccuStandard	MeP-d_4
EtP*	ethyl paraben	120-47-8	AccuStandard	EtP-d_4
PrP*	propyl paraben	94-13-3	AccuStandard	tert-paraben-d_9

BuP*	butyl paraben	94-26-8	AccuStandard	tert-paraben-d_9
BzP	benzyl paraben	94-18-8	AccuStandard	tert-paraben-d_9
HeP	heptyl paraben	1085-12-7	AccuStandard	tert-paraben-d_9
TCC*	triclocarban	101-20-2	Dr. Ehrenstorfer	TCC-$^{13}C_6$
TCS*	triclosan	3380-34-5	AccuStandard	TCC-$^{13}C_6$
Pesticides (PTs)				
ACE	acetamiprid	135410-20-7	Dr. Ehrenstorfer	MCPP-d3
ALC	alachlor	15972-60-8	Dr. Ehrenstorfer	MCPP-d3
AME	ametryn	834-12-8	Dr. Ehrenstorfer	SMZ-d_{10}
ATR	atraton	1610-17-9	Dr. Ehrenstorfer	SMZ-d_{10}
ATZ	atrazine	1912-24-9	Dr. Ehrenstorfer	SMZ-d_{10}
AZX	azoxystrobin	131860-33-8	Dr. Ehrenstorfer	AZX-d_4
BIT	bitertanol	55179-31-2	Dr. Ehrenstorfer	AZX-d_4
BOS	boscalid	188425-85-6	Dr. Ehrenstorfer	BOS-d_4
CBD	carbendazim	10605-21-7	AccuStandard	TBZ-d_4
CBF	carbofuran	1563-66-2	AccuStandard	CBF-d_3
CLL	chlortoluron	15545-48-9	Dr. Ehrenstorfer	MCPP-d_3
CLX	chloroxuron	1982-47-4	Dr. Ehrenstorfer	BOS-d_4
CLZ	chloridazon	1698-60-8	Dr. Ehrenstorfer	BOS-d_4
CMP	coumaphos	56-72-4	Dr. Ehrenstorfer	MCPP-d_3
CPZ	cyproconazole	94361-06-5	Dr. Ehrenstorfer	PPN-d_5
2.4-DC	2,4-dichlorophenol	120-83-2	Dr. Ehrenstorfer	PPN-d_5
2.5-DC	2,5-dichlorophenol	583-78-8	Dr. Ehrenstorfer	PPN-d_5
DCPMU	1-(3,4-dichlorophenyl)-3-methylurea	3567-62-2	Dr. Ehrenstorfer	PPN-d_5
DCPU	3,4-dichlorophenylurea	2327-02-8	Dr. Ehrenstorfer	PPN-d_5
DCP*	dichlorprop	120-36-5	Dr. Ehrenstorfer	PPN-d_5

DCV*	dichlorvos	62-73-7	AccuStandard	PPN-d_5
DFZ	difenoconazole	119446-68-3	Dr. Ehrenstorfer	BOS-d_4
DIN	dinotefuran	165252-70-0	Dr. Ehrenstorfer	CBF-d_3
DMC	dimethachlor	50563-36-5	Dr. Ehrenstorfer	MCPP-d_3
DMO	dimethoate	60-51-5	AccuStandard	TBZ-d_4
DMST	N,N-dimethyl-N'-p-tolylsulphamide	66840-71-9	Dr. Ehrenstorfer	TBZ-d_4
DZN	diazinon	333-41-5	AccuStandard	TBZ-d_4
DUB*	dursban	2921-88-2	AccuStandard	TBZ-d_4
EPZ	epoxiconazole	133855-98-8	AccuStandard	PPN-d_5
FBZ	fenbuconazole	114369-43-6	Dr. Ehrenstorfer	PPN-d_5
FCP	fluacrypyrim	229977-93-9	AccuStandard	AZX-d_4
FEN	fenuron	101-42-8	Dr. Ehrenstorfer	IPR-d_6
FIP	fipronil	120068-37-3	Dr. Ehrenstorfer	PPN-d_5
FMD	famoxadone	131807-57-3	Dr. Ehrenstorfer	AZX-d_4
FNX	fenhexamid	126833-17-8	Dr. Ehrenstorfer	PPN-d_5
4F-3-PBA*	4-fluoro-3-phenoxy benzoic acid	77279-89-1	Dr. Ehrenstorfer	PMN-d_5
FSZ	flusilazole	85509-19-9	Dr. Ehrenstorfer	TBZ-d_4
FTN	flutolanil	66332-96-5	Dr. Ehrenstorfer	PMN-d_5
HEX	hexazinone	51235-04-2	Dr. Ehrenstorfer	CBF-d_3
IMI	imidacloprid	138261-41-3	Dr. Ehrenstorfer	PPN-d_5
IMZ	imazalil	35554-44-0	Dr. Ehrenstorfer	PPN-d_5
IPD	iprodione	36734-19-7	Dr. Ehrenstorfer	PPN-d_5
IPR	isoproturon	34123-59-6	Dr. Ehrenstorfer	IPR-d_6
IPV*	iprovalicarb	140923-17-7	Dr. Ehrenstorfer	IPR-d_6
KXM	kresoxim-methyl	143390-89-0	Dr. Ehrenstorfer	PMN-d_5
LCT	lambda-cyhalothrin	91465-08-6	Dr. Ehrenstorfer	AZX-d_4

LNR	linuron	330-55-2	Dr. Ehrenstorfer	PPN-d_5
MCPP	mecoprop	93-65-2	Dr. Ehrenstorfer	MCPP-d_3
MB	metobromuron	3060-89-7	Dr. Ehrenstorfer	MCPP-d_3
MNP*	3-methyl-4-nitrophenol	2581-34-2	AccuStandard	IPR-d_6
MTB	metribuzin	21087-64-9	Dr. Ehrenstorfer	TBZ-d_4
MTBU	methabenzthiazuron	18691-97-9	Dr. Ehrenstorfer	TBZ-d_4
MTF	methacrifos	62610-77-9	Dr. Ehrenstorfer	TBZ-d_4
MTM	metamitron	41394-05-2	Dr. Ehrenstorfer	PMN-d_5
MTX	metalaxyl	57837-19-1	Dr. Ehrenstorfer	CBF-d_3
MX	metoxuron	19937-59-8	Dr. Ehrenstorfer	MCPP-d_3
MYC	myclobutanil	88671-89-0	Dr. Ehrenstorfer	SMZ-d_{10}
NUA	nuarimol	63284-71-9	Dr. Ehrenstorfer	SMZ-d_{10}
3-PBA*	3-phenoxybenzoic acid	3739-38-6	Dr. Ehrenstorfer	PMN-d_5
PCZ	prochloraz	67747-09-5	Dr. Ehrenstorfer	PPN-d_5
PEN	penconazole	66246-88-6	Dr. Ehrenstorfer	PPN-d_5
PM	prometon	1610-18-0	Dr. Ehrenstorfer	SMZ-d_{10}
PMC	pirimicarb	23103-98-2	Dr. Ehrenstorfer	IPR-d_6
PMN	pyrimethanil	53112-28-0	Dr. Ehrenstorfer	PMN-d_5
PMT	prometryn	7287-19-6	Dr. Ehrenstorfer	TBZ-d_4
PNP*	p-nitrophenol	100-02-7	Dr. Ehrenstorfer	IPR-d_6
PPN	propiconazole	60207-90-1	Dr. Ehrenstorfer	PPN-d_5
PPZ	propazine	139-40-2	Dr. Ehrenstorfer	SMZ-d_{10}
PRP	pyrazophos	13457-18-6	Dr. Ehrenstorfer	TBZ-d_4
PSC	prosulfocarb	52888-80-9	Dr. Ehrenstorfer	TBZ-d_4
PYR	pyraclostrobin	175013-18-0	Dr. Ehrenstorfer	BOS-d_4
SBT	secbumeton	26259-45-0	Dr. Ehrenstorfer	IPR-d_6

SMT	simetryn	1014-70-6	Dr. Ehrenstorfer	SMZ-d_{10}
SMZ	simazine	122-34-9	Dr. Ehrenstorfer	SMZ-d_{10}
2,4,5-T*	2,4,5-trichlorophenoxyacetic acid	93-76-5	Dr. Ehrenstorfer	MCPP-d_3
TB	terbutryn	886-50-0	Dr. Ehrenstorfer	SMZ-d_{10}
TBZ*	thiabendazole	148-79-8	Dr. Ehrenstorfer	TBZ-d_4
TEZ	tebuconazole	107534-96-3	Dr. Ehrenstorfer	MCPP-d_3
TFS	trifloxystrobin	141517-21-7	Dr. Ehrenstorfer	AZX-d_4
THI	thiacloprid	111988-49-9	Dr. Ehrenstorfer	TBZ-d_4
THX*	thiamethoxam	153719-23-4	Dr. Ehrenstorfer	TBZ-d_4
TTZ	tetraconazole	112281-77-3	Dr. Ehrenstorfer	PPN-d_5
ZPM	pyriminobac-methyl(Z)	147411-70-9	Dr. Ehrenstorfer	AZX-d_4
Photoinitiator additives (PIs)				
BP	benzophenone	119-61-9	TRC	BP-d_{10}
4-MBP	4-methylbenzophenone	134-84-9	TRC	BP-d_{10}
PHBP	4-phenylbenzophenone	2128-93-0	TRC	BP-d_{10}
DMAB	4-(dimethylamino)benzophenone	530-44-9	TRC	BP-d_{10}
MK*	4,4'-bis(dimethylamino)benzophenone	90-94-8	TRC	BP-d_{10}
MEK	4,4'-bis(diethylamino)benzophenone	90-93-7	TRC	BP-d_{10}
MBB	methyl-2-(benzoyl)benzoate	606-28-0	TRC	BP-d_{10}
EAB	ethyl-4-aminobenzoate	94-09-7	TRC	BP-d_{10}
EDMAB	ethyl-4-dimethylaminobenzoate	10287-53-3	TRC	BP-d_{10}
EAQ	2-ethylanthraquinone	84-51-5	TRC	BP-d_{10}
PI-184	1-hydroxycyclohexyl phenyl ketone	947-19-3	TRC	BP-d_{10}
PI-651*	2,2-dimethoxy-2-phenylacetophenone	24650-42-8	TRC	BP-d_{10}
DETX	2,4-diethyl-9H-thioxanthen-9-one	82799-44-8	TRC	BP-d_{10}
2-ITX	2-isopropylthioxanthone	5495-84-1	TRC	BP-d_{10}

Benzil	1,2-diphenyl-1,2-ethanedione	134-81-6	TRC	BP-d_{10}
Mono-phthalate esters (mono-PAEs)				
mMP*	monomethyl phthalate	4376-18-5	AccuStandard	MEPA-d_4
mETP*	monoethyl phthalate	2306-33-4	AccuStandard	MEPA-d_4
miPrP	monoisopropyl phthalate	35118-50-4	AccuStandard	MEPA-d_4
mCPP	mono (3-carboxypropyl) phthalate	66851-46-5	TRC	MEPA-d_4
mBP*	monobutyl phthalate	131-70-4	AccuStandard	MBPA-d_4
miBP*	monoisobutyl phthalate	30833-53-5	AccuStandard	MBPA-d_4
mPeP	mono-n-pentyl phthalate	24539-56-8	AccuStandard	MBPA-d_4
mHxP	monohexyl phthalate	24539-57-9	AccuStandard	MBPA-d_4
mCHP*	monocyclohexyl phthalate	7517-36-4	AccuStandard	MBPA-d_4
mHeP	mono-2-heptyl phthalate	129171-03-5	AccuStandard	MBPA-d_4
mOcP*	monooctyl phthalate	5393-19-1	AccuStandard	MEHPA-d_4
mCiOP	mono-carboxy-isooctyl phthalate	898544-09-7	TRC	MEHPA-d_4
miNoP	monoisononyl phthalate	106610-61-1	AccuStandard	MEHPA-d_4
mHiNP*	monohydroxyisononyl phthalate	898544-10-0	TRC	MEHPA-d_4
mCDP	monocarboxyisodecyl phthalate	NA[b]	TRC	MEHPA-d_4
mBzP*	monobenzyl phthalate	2528-16-7	AccuStandard	MBzPA-d_4
mEHP*	monoethylhexyl phthalate	4376-20-9	AccuStandard	MEHPA-d_4
mEHHP*	mono (2-ethyl-5-hydroxyhexyl) phthalate	40321-99-1	TRC	MEHPA-d_4
mEOHP*	mono (2-ethyl-5-oxohexyl) phthalate	40321-98-0	TRC	MEHPA-d_4
mECPP*	mono (2-ethyl-5carboxypentyl) phthalate	40809-41-4	TRC	MEHPA-d_4
mCMHP*	mono[2-(carboxymethyl)hexyl] phthalate	82975-93-7	AccuStandard	MEHPA-d_4
Non-PAE plasticizers (NPPs)				
DMAZ	dimethyl azelate	1732-10-1	AccuStandard	ATBC-d_3
DHAZ	di-n-hexyl azelate	109-31-9	AccuStandard	ATBC-d_3

DiOAZ	diisooctyl azelate	26544-17-2	AccuStandard	ATBC-d_3
DiDeAZ	diisodecyl azelate	28472-97-1	AccuStandard	ATBC-d_3
DEGDB	diethylene glycol, dibenzoate	120-55-8	AccuStandard	ATBC-d_3
DPGDB	dipropylene glycol, dibenzoate	27138-31-4	AccuStandard	ATBC-d_3
TPIB	2,2,4-trimethyl-1,3-pentanediol-monoisobutyrate	25265-77-4	AccuStandard	ATBC-d_3
TXIB*	2,2,4-trimethyl-1,3-pentanediol-diisobutyrate	6846-50-0	AccuStandard	ATBC-d_3
TEC	triethyl citrate	77-93-0	AccuStandard	ATBC-d_3
TBC*	tributyl citrate	77-94-1	AccuStandard	ATBC-d_3
ATEC	acetyl triethyl citrate	77-89-4	AccuStandard	ATBC-d_3
ATBC*	acetyl tri-n-butyl citrate	77-90-7	AccuStandard	ATBC-d_3
DEHM*	di(2-ethylhexyl) maleate	142-16-5	AccuStandard	ATBC-d_3
BARO	butyl acetyl ricinoleate	140-04-5	AccuStandard	ATBC-d_3
BO	butyl oleate	142-77-8	AccuStandard	ATBC-d_3
GMO	glycerol monooleate	25496-72-4	AccuStandard	ATBC-d_3
MO	methyl oleate	112-62-9	AccuStandard	ATBC-d_3
PO	n-propyl oleate	111-59-1	AccuStandard	ATBC-d_3
THFO	tetrahydrofurfuryl oleate	5420-17-7	AccuStandard	ATBC-d_3
DMS	dimethyl sebacate	106-79-6	AccuStandard	ATBC-d_3
DBS	dibutyl sebacate	109-43-3	AccuStandard	ATBC-d_3
EHSe	2-ethylhexyl sebacate	122-62-3	AccuStandard	ATBC-d_3
GMS	glycerol monostearate	31566-31-1	AccuStandard	ATBC-d_3
DES	diethyl succinate	123-25-1	AccuStandard	ATBC-d_3
DEHT	bis(2-ethylhexyl) terephthalate	6422-86-2	AccuStandard	ATBC-d_3
Synthetic antioxidants (SAOs)				
AO425	2,2'-methylenebis(4-ethyl-6-tert-butylphenol)	88-24-4	AccuStandard	MBPA-d_4
AO1222	diethyl-3,5-di-tert-butyl-4-hydroxybenzyl phosphonate	976-56-7	AccuStandard	MBPA-d_4

AO2246*	2,2'-methylenebis(6-tert-butyl-4-methylphenol)	119-47-1	AccuStandard	MBPA-d_4
AO22E46	2,2'-ethylidene-bis(4,6-di-tert-butylphenol)	35958-30-6	AccuStandard	MBPA-d_4
BBOT	2,2'-(2,5-thiophenediyl)-bis (5-tert-butylbenzoxazole)	7128-64-5	AccuStandard	MBPA-d_4
BHA*	3-tert-butyl-4-hydroxyanisole	121-00-6	AccuStandard	tert-paraben-d_9
BHT*	2,6-di-tert-butyl-4-hydroxytoluene	128-37-0	AccuStandard	tert-paraben-d_9
BHT-CHO*	3,5-di-tert-butyl-4-hydroxybenzaldehyde	1620-98-0	AccuStandard	tert-paraben-d_9
BHT-COOH*	3,5-di-tert-butyl-4-hydroxybenzoic acid	1421-49-4	TCI	tert-paraben-d_9
BHT-OH*	2,6-di-tert-butyl-4-(hydroxymethyl)phenol	88-26-6	AccuStandard	tert-paraben-d_9
BHT-Q*	2,6-di-tert-butyl-1,4-benzoquinone	719-22-2	TRC	tert-paraben-d_9
BHT-quinol*	2,6-di-tert-butyl-4-hydroxy-4-methyl-2,5-cyclohexadienone	10396-80-2	TRC	tert-paraben-d_9
2,4DtBP*	2,4-di-tert-butylphenol	96-76-4	AccuStandard	tert-paraben-d_9
4-tOP*	4-(1,1,3,3-tetra-methylbutyl)phenol	140-66-9	AccuStandard	tert-paraben-d_9
DBHA	dibenzylhydroxylamine	621-07-8	AccuStandard	tert-paraben-d_9
DPG	1,3-diphenylguanidine	102-06-7	AccuStandard	tert-paraben-d_9
DPPD	N,N'-diphenyl-1,4-benzenediamine	74-31-7	AccuStandard	tert-paraben-d_9
DTG	1,3-di-o-tolylguanidine	97-39-2	AccuStandard	tert-paraben-d_9
Ethanox 703	2,6-di-tert-butyl-4-(dimethylamino-methyl)phenol	88-27-7	AccuStandard	MBPA-d_4
Irganox 245	triethylene glycol bis(3-tert-butyl-4-hydroxy-5-methylphenyl)propionate	36443-68-2	AccuStandard	MBPA-d_4
Irganox 259	benzenepropanoic acid, 3,5-bis(1,1-dimethylethyl)-4-hydroxy-, 1,6-hexanediyl ester	35074-77-2	AccuStandard	MBPA-d_4
Irganox 1035	thiodiethylene bis(3-(3,5-di-tert-butyl-4-hydroxyphenyl)propionate)	41484-35-9	AccuStandard	MBPA-d_4
Irganox 1081	2,2'-thiobis(6-tert-butyl-p-cresol)	90-66-4	AccuStandard	MBPA-d_4
Irganox 1098	N,N'-1,6-hexanediylbis{3-[4-hydroxy-3,5-bis(2-methyl-2-propanyl)phenyl]propanamide}	23128-74-7	AccuStandard	MBPA-d_4

NPN	N-phenyl-1-naphthylamine	90-30-2	AccuStandard	MBPA-d_4
UV stabilizers (UVs)				
BP-1*	2,4-dihydroxybenzophenone	131-56-6	AccuStandard	BP-d_{10}
BP-2	2,2',4,4'-tetrahydroxybenzophenone	131-55-5	Sigma-Aldrich	BP-d_{10}
BP-3*	2-hydroxy-4-methoxybenzophenone	131-57-7	AccuStandard	BP-d_{10}
BP-4*	2-hydroxy-4-methoxybenzophenone-5-sulfonic acid hydrate	4065-45-6	TRC	BP-d_{10}
BP-6	2,2-dihydroxy-4,4-dimethoxybenzophenone	131-54-4	AccuStandard	BP-d_{10}
BP-8	2,2′-dihydroxy-4-methoxybenzophenone	131-53-3	TRC	BP-d_{10}
BP-12	2-hydroxy-4-(octyloxy)benzophenone	1843-05-6	AccuStandard	BP-d_{10}
4-OH-BP*	4-hydroxybenzophenone	1137-42-4	Sigma-Aldrich	2,4-OH-BP-$^{13}C_6$
1-H-BTR	1-hydrogen-benzotriazole	95-14-7	Chemservice	1-H-BTR-d_4
1-OH-BTR	1-hydroxybenzotriazole	2592-95-2	TCI	1-H-BTR-d_4
5-Cl-BTR	5-chloro-1-hydrogenbenzotriazole	94-97-3	Santa Cruz	1-H-BTR-d_4
4-Me-1-H-BTR	4-methyl-1-hydrogenbenzotriazole	29878-31-7	Santa Cruz	5-Me-BTR-d_6
5-Me-1-H-BTR	5-methyl-1-hydrogenbenzotriazole	136-85-6	Chemservice	5-Me-BTR-d_6
5,6-2Me-1-HBTR	5,6-dimethyl-1-hydrogenbenzotriazole	4184-79-6	TCI	5-Me-BTR-d_6
BTH	benzothiazole	95-16-9	Sigma-Aldrich	BTH-d_4
2-OH-BTH	2-hydroxybenzothiazole	934-34-9	Chemservice	BTH-d_4
2-Cl-BTH	2-chlorobenzothiazole	615-20-3	TCI	BTH-d_4
2-Me-BTH	2-methylbenzothiazole	120-75-2	TCI	BTH-d_4
2-Me-S-BTH	2-(methylthio)benzothiazole	615-22-5	TCI	BTH-d_4
2-Mo-BTH	2-(morpholinothio)-benzothiazole	102-77-2	TCI	BTH-d_4
2NH_2-BTH*	2-aminobenzothiazole	136-95-8	TRC	BTH-d_4
EHS	2-ethylhexyl salicylate	118-60-5	AccuStandard	5-Me-BTR-d_6
HMS	3,3,5-trimethylcyclohexyl salicylate	118-56-9	AccuStandard	5-Me-BTR-d_6
IAMC	isoamyl 4-methoxycinnamate	71617-10-2	TCI	5-Me-BTR-d_6

4-MBC	4-methylbenzylidene camphor	36861-47-9	AccuStandard	5-Me-BTR-d_6
ODPABA	octyl dimethyl-p-aminobenzoic acid	21245-02-3	AccuStandard	5-Me-BTR-d_6
OMC	ethylhexyl methoxycinnamate	5466-77-3	AccuStandard	5-Me-BTR-d_6
UV-P	2-(2-hydroxy-5-methylphenyl)benzotriazole	2440-22-4	AccuStandard	5-Me-BTR-d_6
UV-PS	2-(5-tert-butyl-2-hydroxyphenyl) benzotriazole	3147-76-0	AccuStandard	5-Me-BTR-d_6
UV-234	2-(2H-benzotriazol-2-yl)-4,6-bis(1-methyl-1-phenylethyl) phenol	70321-86-7	AccuStandard	5-Me-BTR-d_6
UV-320	2-(3,5-di-tert-butyl-2-hydroxyphenyl) 2H-benzotriazole	3846-71-7	AccuStandard	5-Me-BTR-d_6
UV-326	2-tert-butyl-6-(5-chloro-2H-benzotriazol-2-yl)-4-methylphenol	96-11-5	AccuStandard	5-Me-BTR-d_6
UV-327	2,4-di-tert-butyl-6-(5-chloro-2H-benzotriazol-2-yl) phenol	3864-99-1	AccuStandard	5-Me-BTR-d_6
UV-328	2-(2H-benzotriazol-2-yl)-4,6-di-tert-pentylphenol	25973-55-1	AccuStandard	5-Me-BTR-d_6
UV-329	2-(2'-hydroxy-3',5'-di-tert-amylphenyl)benzotriazole	3147-75-9	AccuStandard	5-Me-BTR-d_6
UV-350	2-(3-sec-butyl-5-tert-butyl-2-hydroxyphenyl) benzotriazole	36437-37-3	AccuStandard	5-Me-BTR-d_6
UV-360	bisoctrizole	103597-45-1	TRC	5-Me-BTR-d_6
**Internal standards**				
BPA-d_{16}	bisphenol A-d_{16}	96210-87-6	Sigma-Aldrich	
CMP-d_{10}	coumaphos-d_{10}	287397-86-8	TRC	
TCC-$^{13}C_6$	triclocarban-$^{13}C_6$	1216457-76-9	TRC	
**Surrogate standards**				
2-OH-Nap-d_8	2-hydroxynaphthalene-d_8	78832-61-8	TRC	
3-OH-Flu-d_9	3-hydroxyfluorene-d_9	N/A	TRC	
1-OH-Pyr-d_9	1-hydroxypyrene-d_9	132603-37-3	TRC	
3-OH-Phe-$^{13}C_4$	9-hydroxyphenanthrene-$^{13}C_4$		TRC	
TEP-d_{15}	triethyl phosphate-d_{15}	135942-11-9	Wellington	
TBP-d_{27}	tri-n-butyl phosphate-d_{27}	61196-26-7	Wellington	
TPHP-d_{15}	triphenyl phosphate-d_{15}	1173020-30-8	Wellington	

M6TBOEP	tris(2-butoxy-[13C2]-ethyl) phosphate	NA	Wellington
TCEP-d_{12}	tris(2-chloroethyl) phosphate-d_{12}	1276500-47-0	Wellington
TDCIPP-d_{15}	tris(1,3-dichloro-2-propyl) phosphate-d_{15}	1447569-77-8	Wellington
BDCIPP-d_{10}	bis(1,3-dichloro-2-propyl) phosphate-d_{10}	1477495-19-4	Wellington
BCEP-d_8	bis(2-chloroethyl) phosphate-d_8	1477495-02-5	TRC
BCIPP-d_{12}	bis-(1-chloro-2-propyl) phosphate-d_{12}	NA	TRC
BBOEP-d_8	bis(butoxyethyl) phosphate-d_8	NA	TRC
DoCP-d_{14}	di-o-cresyl phosphate-d_{14}	NA	TRC
DpCP-d_{14}	di-p-tolyl-phosphate-d_{14}	NA	TRC
DBP-d_{18}	dibutyl phosphate-d_{18}	156213-21-7	TRC
BEHP-d_{34}	bis(2-ethylhexyl) phosphate -d_{34}	1773493-20-1	TRC
BTH-d_4	benzothiazole-d_4	194423-51-3	TRC
MBT-d_4	2-benzothiazolethiol-d_4	1346598-64-8	TRC
1-H-BTR-d_4	1H-benzotriazole-d_4	1185072-03-0	TRC
5-Me-BTR-d_6	5-methyl-benzotriazole-d_6	1246820-65-4	TRC
BHT-d_{21}	2,6-di(tert-butyl)-4-methyl-phenol-d_{21}	64502-99-4	Dr. Ehrenstorfer
ATBC-d_3	tributyl O-acetylcitrate-d_3	1794753-49-3	TRC
MEPA-d_4	monoethyl phthalate-d_4	1219806-03-7	TRC
MBPA-d_4	mono-n-butyl phthalate-d_4	478954-81-3	CDN Isotopes
MBzPA-d_4	mono-benzyl phthalate-d_4	478954-83-5	CDN Isotopes
MEHPA-d_4	rac mono(ethylhexyl) phthalate-d_4	1276197-22-8	TRC
BP-d_{10}	benzophenone-d_{10}	22583-75-1	CDN Isotopes
2,4-OH-BP-$^{13}C_6$	2,4-dihydroxybenzophenone-$^{13}C_6$	2731164-01-3	TRC
MeP-d_4	methyl paraben-d_4	362049-51-2	TRC
EtP-d_4	ethyl paraben-d_4	1219795-53-5	TRC
tert-paraben-d_9	tert-paraben-d_9	1216904-65-2	TRC

TCS-d_3	triclosan-d_3	1020719-98-5	TRC
AZX-d_4	azoxystrobin-d_4	1346606-39-0	TRC
BOS-d_4	boscalid-d_4	NA	TRC
CBF-d_3	carbofuran-d_3	1007459-98-4	TRC
IPR-d_6	isoproturon-d_6	1007461-76-8	Dr. Ehrenstorfer
MCPP-d_3	mecoprop-d_3	352431-15-3	Dr. Ehrenstorfer
PMN-d_5	pyrimethanil-d_5	NA	TRC
PPN-d_5	propiconazole-d_5	NA	Dr. Ehrenstorfer
SMZ-d_{10}	simazine-d_{10}	220621-39-6	Dr. Ehrenstorfer
TBZ-d_4	thiabendazole-d_4	1190007-20-5	TRC

Notes: * Selected for protocol development and quality assurance and control tests;

[a] NA = not available;

[b] Suppliers—AccuStandard: AccuStandard Inc. (New Haven, Connecticut, USA); Dr. Ehrenstorfer: LGC Standards (Middlesex, UK); Sigma-Aldrich: Sigma-Aldrich, Inc. (St. Louis, Missouri, USA); TCI: TCI America (Seekonk, Massachusetts, USA); TRC: Toronto Research Chemicals (Toronto, Canada); Wellington: Wellington Laboratories (Guelph, Ontario, Canada).

Table S4. Major chemical-dependent parameters for instrumental analysis. They include the multiple-reaction-monitoring (MRM) ion transitions, declustering potential (DP), and collision energy (CE) of individual exogenous chemicals, as well as the description of the corresponding instrumental methods.

Commercial name or abbreviation	Quantitative ion			Qualitative ion			Instrumental method[a]
	Ion transition	DP	CE	Ion transition	DP	CE	
**Target exogenous chemicals**							
Organophosphate esters (OPEs)							
TEP	183.1→99.0		24	183.1→81.0		52	M1
TPrP	225.1→98.9		28	225.1→80.9		61	M1
TBP	267.1→98.9		23	267.1→80.9		70	M1
TPHP	327.1→77.1		64	327.1→152.1		52	M1
TCrP	369.1→165.0		62	369.1→91.0		62	M1
TEHP	435.3→99.0		30	435.3→81.0		30	M1
TBOEP	399.1→199.1		20	399.1→101.0		20	M1
TCEP	284.9→99.0		32	284.9→62.9		44	M1
TCIPP	329.0→99.0		33	329.0→81.0		89	M1
TDCIPP	430.9→99.0		34	430.9→81.0		102	M1
TDBPP	698.4→98.9		85	698.4→118.9		145	M1
T34DMPP	411.2→178.8		51	411.2→194.2		41	M1
T35DMPP	411.1→179.1		51	411.1→194.0		42	M1
BPADP	693.0→367.2		48	693.0→178.2		78	M1
CDP	340.9→151.9		51	340.9→91.1		51	M1

EHDPHP	363.2→250.9	15	363.2→76.9	63	M1
IDDPP	391.0→251.1	21	391.0→77.1	76	M1
RDP	575.0→77.1	124	575.0→152.2	110	M1
V6	582.9→99.1	73	582.9→65.1	104	M1
2IPPDPP	369.2→327.0	27	369.2→233.0	37	M1
4IPPDPP	369.2→327.0	29	369.2→233.0	39	M1
24DIPPDPP	411.2→369.0	24	411.2→327.0	35	M1
B2IPPPP	411.3→369.0	23	411.3→327.0	34	M1
B4IPPPP	411.3→369.1	27	411.3→327.0	37	M1
B24DIPPPP	495.3→411.0	35	495.3→369.0	42	M1
T2IPPP	452.9→326.9	41	452.9→91.0	93	M1
T3IPPP	453.2→411.3	30	453.2→369.3	38	M1
T4IPPP	453.3→411.2	30	453.3→369.2	38	M1
2tBPDPP	383.1→327.0	26	383.1→251.0	42	M1
4tBPDPP	383.2→327.0	28	383.2→233.0	47	M1
B2tBPPP	439.3→383.2	21	439.3→327.0	24	M1
B4tBPPP	439.3→383.0	47	439.3→327.0	47	M1
T4tBPP	495.3→383.0	38	495.3→439.0	32	M1
DMP	125.0→79.0	-36	125.0→78.9	-40	M1
DEP	153.0→78.9	-32	152.6→79.0	-30	M1
DBP	208.9→78.8	-80	208.9→153.2	-20	M1
DPHP	249.0→155.1	-28	249.0→92.9	-34	M1
DoCP	277.0→107.0	-32	277.0→78.8	-70	M1
DpCP	277.0→107.0	-32	277.0→78.8	-70	M1
BEHP	321.1→78.9	-35	321.1→208.9	-30	M1
BBOEP	297.0→79.0	-66	297.0→71.0	-66	M1

BCEP	222.8→37.0	-20	222.8→35.0	-20	M1
BCIPP	248.8→35.0	-23	248.8→113.0	-12	M1
BDCIPP	319.0→35.0	-35	319.0→37.1	-47	M1
BDBPP	496.8→80.9	-60	496.8→78.9	-50	M1
Phenolic polycyclic aromatic hydrocarbons (OH-PAHs)					
1-OH-Nap	143.0→115.0	-34	143.0→142.0	-34	M2
2-OH-Nap	143.0→115.0	-34	143.0→142.0	-34	M2
2-OH-Flu	181.0→180.0	-32	181.0→152.9	-32	M2
3-OH-Flu	181.0→180.0	-32	181.0→152.9	-32	M2
9-OH-Flu	181.0→180.0	-32	181.0→152.9	-32	M2
3-OH-Fla	172.7→145.0	-25	172.7→117.0	-40	M2
1-OH-Pyr	217.0→189.0	-47	217.0→188.0	-53	M2
1-OH-Phe	193.0→165.1	-40	193.0→192.0	-40	M2
2-OH-Phe	193.0→165.1	-40	193.0→192.0	-40	M2
3-OH-Phe	193.0→165.1	-40	193.0→192.0	-40	M2
4-OH-Phe	193.0→165.1	-40	193.0→192.0	-40	M2
9-OH-Phe	193.0→165.1	-40	193.0→192.0	-40	M2
3-OH-BaP	238.9→223.0	-24	238.9→75.0	-43	M2
3-OH-BcP	243.0→215.0	-50	243.0→241.0	-50	M2
6-OH-Chr	243.0→215.0	-50	243.0→241.0	-50	M2
Personal care products (PCPs)					
MeP	151.0→92.0	-26	151.0→135.9	-18	M1
EtP	165.0→91.9	-30	165.0→136.9	-18	M1
PrP	179.1→92.0	-33	179.1→136.0	-21	M1
BuP	193.3→92.0	-31	193.3→93.0	-28	M1
BzP	227.1→92.1	-32	227.1→136.0	-20	M1

HeP	235.1→93.0	-31	235.1→136.0	-25	M1
TCC	314.8→161.9	-24	314.8→159.7	-27	M1
TCS	286.8→35.0	-35	286.8→142.1	-29	M1
Pesticides (PTs)					
ACE	223.2→126.1	31	223.2→99.1	54	M1
ALC	270.0→238.0	17	270.0→162.0	23	M1
AME	228.1→186.1	27	228.1→157.8	35	M1
ATR	212.2→170.1	27	212.2→142.0	34	M1
ATZ	216.1→174.1	25	216.1→146.2	31	M1
AZX	404.1→344.1	34	404.1→372.1	19	M1
BIT	338.0→269.0	15			M1
BOS	343.0→307.0	25	343.0→140.0	21	M1
CBD	192.2→160.2	15	192.2→132.1	39	M1
CBF	222.2→123.1	26	222.2→104.1	29	M1
CLL	213.1→72.0	36	213.1→140.1	32	M1
CLX	291.1→164.3	25	291.1→218.1	36	M1
CLZ	222.2→104.1	29	222.2→128.9	31	M1
CMP	363.0→227.0	41	363.0→307.0	25	M1
CPZ	292.2→125.2	45	292.2→70.2	42	M1
2.4-DC	161.0→125.0	-22	161.0→89.0	-30	M1
2.5-DC	161.0→125.0	-22	161.0→89.0	-30	M1
DCPMU	219.2→162.2	20			M1
DCPU	205.0→127.0	34	205.0→162.0	22	M1
DCP	234.9→162.9	-17	232.9→160.9	-21	M1
DCV	221.2→109.0	23	221.2→145.1	18	M1
DFZ	406.1→251.0	34	406.1→337.0	28	M1

DIN	203.1→157.0	12	203.1→129.0	15	M1
DMC	256.0→224.1	24	256.0→148.1	36	M1
DMO	230.3→198.8	10	230.3→125.0	28	M1
DMST	215.0→106.0	19	215.0→151.0	9	M1
DZN	305.1→153.0	28	305.1→169.1	26	M1
DUB	349.9→198.0	21	349.9→97.0	39	M1
EPZ	330.1→121.0	29	330.1→295.0	14	M1
FBZ	337.1→125.0	37	337.1→70.0	33	M1
FCP	427.1→145.1	41	427.1→205.1	18	M1
FEN	165.1→72.0	15	165.1→120.0	23	M1
FIP	437.2→368.0	23	437.2→290.1	36	M1
FMD	392.0→238.0	23	392.0→331.0	12	M1
FNX	302.0→97.1	27			M1
4F-3-PBA	231.1→93.1	-30	231.1→187.0	-16	M1
FSZ	316.1→247.1	23	316.1→165.1	36	M1
FTN	324.1→282.2	20			M1
HEX	253.0→171.0	18	253.0→71.0	42	M1
IMI	256.2→209.0	23	256.2→175.2	23	M1
IMZ	297.1→159.2	29	297.1→201.0	28	M1
IPD	330.1→245.0	18	330.1→288.0	16	M1
IPR	206.8→72.0	17	206.8→165.3	23	M1
IPV	321.0→119.0	30	321.0→203.0	9	M1
KXM	314.1→267.1	8	314.1→206.2	8	M1
LCT	467.3→225.2	19			M1
LNR	249.0→160.0	23	249.0→182.0	20	M1
MCPP	213.0→140.6	-17			M1

MB	259.0→170.0	23	259.0→148.0	26	M1
MNP	151.9→122.1	-23	151.9→107.1	-23	M1
MTB	215.1→187.2	27	215.1→84.1	27	M1
MTBU	222.0→150.0	40			M1
MTF	241.2→125.1	26	241.2→209.1	10	M1
MTM	203.3→175.1	20	203.3→104.1	28	M1
MTX	279.8→220.0	15	279.8→248.0	15	M1
MX	229.3→72.1	21	229.3→156.1	32	M1
MYC	289.1→125.0	48	289.1→70.0	24	M1
NUA	315.1→252.1	36	315.1→243.0	42	M1
3-PBA	213.4→93.1	-27			M1
PCZ	376.1→308.0	19	376.1→266.0	19	M1
PEN	284.0→159.0	46	284.0→70.0	20	M1
PM	226.2→184.1	27			M1
PMC	239.2→182.2	24	239.2→195.3	15	M1
PMN	200.0→181.0	34	200.0→103.0	34	M1
PMT	242.1→200.1	27			M1
PNP	138.2→107.9	-23	138.2→92.0	-27	M1
PPN	342.0→154.0	32	342.0→182.0	32	M1
PPZ	230.1→188.1	27			M1
PRP	374.3→222.3	25	374.3→238.1	25	M1
PSC	252.4→91.2	23	252.4→128.2	16	M1
PYR	388.1→193.8	16	388.1→296.0	15	M1
SBT	226.2→170.1	27			M1
SMT	214.1→124.1	30	214.1→144.0	29	M1
SMZ	202.1→132.0	27	202.1→174.1	25	M1

2,4,5-T	254.9→196.9	-14.13	254.9→194.9	-14.2	M1
TB	242.1→186.1	27			M1
TBZ	202.1→175.1	38	202.1→131.2	38	M1
TEZ	308.0→70.0	39	308.0→125.0	47	M1
TFS	409.3→186.0	21	409.3→206.2	18	M1
THI	253.1→126.1	25	253.1→186.0	19	M1
THX	292.0→211.0	14	292.0→181.0	27	M1
TTZ	372.0→159.0	31	372.0→70.0	27	M1
ZPM	362.3→330.3	20	362.3→298.2	26	M1
Photoinitiator additives (PIs)					
BP	183.1→105.1	22	183.1→77.1	44	M1
4-MBP	197.2→105.1	25	197.2→119.0	21	M1
PHBP	259.2→105.1	26	259.2→77.2	55	M1
DMAB	226.3→105.1	27	226.3→77.1	55	M1
MK	269.3→148.1	34	269.3→120.1	43	M1
MEK	325.4→176.1	38	325.4→133.1	62	M1
MBB	241.2→209.1	20	241.2→152.2	50	M1
EAB	166.2→138.1	15	166.2→127.1	38	M1
EDMAB	194.3→151.2	32	194.3→166.3	23	M1
EAQ	237.2→209.2	30	237.2→153.0	38	M1
PI-184	205.2→105.1	18	205.2→187.1	9	M1
PI-651	225.2→197.1	21	225.2→105.1	30	M1
DETX	269.3→241.1	30	269.3→213.0	39	M1
2-ITX	255.3→213.1	30	255.3→184.0	53	M1
Benzil	211.2→105.0	15	211.2→77.1	48	M1
Mono-phthalate esters (mono-PAEs)					

MMPA	179.0→77.1	-24	179.0→107.0	-14	M1
MEPA	193.0→77.0	-24	193.0→121.0	-18	M1
MiPrPA	207.1→77.0	-22	207.1→120.9	-20	M1
MCPPA	251.0→102.8	-12	251.0→165.0	-14	M1
MBPA	221.0→77.0	-24	221.0→134.0	-20	M1
MiBPA	221.0→77.0	-24	221.0→134.0	-20	M1
MPePA	235.0→77.0	-26	235.0→85.0	-20	M1
MHxPA	249.0→77.0	-30	249.0→99.1	-22	M1
MCHPA	247.0→77.0	-30	247.0→97.0	-24	M1
MHePA	263.0→77.0	-34	263.0→113.1	-20	M1
MOcPA	277.0→77.1	-34	277.0→127.1	-24	M1
MCiOPA	321.2→161.9	-16	321.2→160.1	-27	M1
MiNoPA	291.0→77.0	-36	291.0→141.1	-26	M1
MHiNPA	307.3→120.9	-28	307.3→77.0	-40	M1
MCDPA	337.0→167.0	29	337.0→76.9	92	M1
MBzPA	255.1→77.0	-30	255.1→107.0	-20	M1
MEHPA	277.0→77.0	-36	277.0→134.1	-22	M1
MEHHPA	293.1→121.1	-21	293.1→144.7	-21	M1
MEOHPA	291.0→121.1	-24	291.0→143.1	-16	M1
MECPPA	306.9→159.1	-20	306.9→113.1	-35	M1
MCMHPA	307.0→113.1	-25	307.0→159.1	-11	M1
Non-PAE plasticizers (NPPs)					
DMAZ	217.1→185.2	10	217.1→97.1	20	M1
DHAZ	357.5→171.1	20	357.5→255.2	14	M1
DiOAZ	413.5→283.2	13	413.5→171.1	18	M1
DiDeAZ	469.0→170.9	24	469.0→71.2	25	M1

DEGDB	315.3→149.1	41	315.3→105.1	12	M1
DPGDB	343.3→163.1	11	343.3→105.1	37	M1
TPIB	217.4→69.1	20	217.4→111.1	11	M1
TXIB	287.4→111.2	16	287.4→199.2	7	M1
TEC	277.3→157.1	19	277.3→203.1	11	M1
TBC	361.5→185.1	18	361.5→129.1	27	M1
ATEC	319.3→157.2	25	319.3→273.1	9	M1
ATBC	403.2→185.2	25	403.2→128.9	35	M1
DEHM	341.4→117.1	13	341.4→99.1	37	M1
BARO	397.5→337.1	9	397.5→263.2	13	M1
BO	339.5→265.2	15	339.5→247.2	18	M1
GMO	357.4→265.4	13	357.4→247.3	17	M1
MO	297.4→247.1	15	297.4→265.3	11	M1
PO	325.4→265.1	15	325.4→247.3	17	M1
THFO	367.4→85.1	16	367.4→265.3	16	M1
DMS	231.2→199.3	11	231.2→139.3	20	M1
DBS	315.4→241.2	19	315.4→185.2	12	M1
EHSe	427.5→185.1	18	427.5→315.3	11	M1
GMS	359.4→341.3	12	359.4→267.0	15	M1
DES	175.2→129.1	11	175.2→101.1	17	M1
DEHT	391.4→149.1	23	391.4→71.2	23	M1
Synthetic antioxidants (SAOs)					
AO425	367.2→177.2	-30	367.2→162.0	-40	M1
AO1222	355.2→137.0	-30	355.2→108.1	-30	M1
AO2246	339.1→163.2	-40	339.1→147.2	-50	M1
AO22E46	456.5→233.2	19	456.5→177.1	39	M1

BBOT	431.2→399.0	80	431.2→385.2	80	M1
BHA	179.0→149.0	-28	179.0→107.7	-42	M1
BHT	219.1→147.0	-34	219.1→133.0	-38	M1
BHT-CHO	235.0→179.1	19	235.0→57.0	81	M1
BHT-COOH	249.0→205.2	-30	249.0→189.0	-40	M1
BHT-OH	235.1→160.1	-40	234.9→160.1	-35	M1
BHT-Q	220.1→205.1	-33	220.1→148.0	-41	M1
BHT-quinol	235.1→220.1	-24	235.1→205.1	-40	M1
2,4DtBP	205.3→189.2	-35	205.1→189.1	-35	M1
4-tOP	205.2→133.1	-30	205.1→93.0	-62	M1
DBHA	214.2→106.1	15	214.2→90.9	16	M1
DPG	212.2→119.1	27	212.2→94.2	26	M1
DPPD	261.2→184.2	35	261.2→169.2	30	M1
DTG	240.1→133.2	28	240.1→108.1	28	M1
Ethanox 703	264.2→219.2	24	264.2→203.1	38	M1
Irganox 245	587.5→177.2	71	587.5→263.0	29	M1
Irganox 259	656.5→471.3	25	656.5→415.1	34	M1
Irganox 1035	660.5→193.0	61	660.5→309.2	31	M1
Irganox 1081	357.1→193.9	-50	357.1→163.2	-50	M1
Irganox 1098	637.5→525.4	35	637.5→581.4	31	M1
NPN	220.1→142.0	55	220.1→115.1	31	M1
UV stabilizers (UVs)					
BP-1	213.0→91.0	-35	213.0→135.0	-25	M1
BP-2	245.0→135.0	-18	245.0→109.0	-25	M1
BP-3	227.0→211.0	-50	227.0→212.0	-25	M1
BP-4	307.0→227.0	-36	307.0→211.0	-46	M1

BP-6	259.0→109.0	-23	259.0→135.0	-16	M1
BP-8	243.0→93.0	-27	243.0→123.0	-22	M1
BP-12	325.0→164.0	-14	325.0→134.0	-44	M1
4-OH-BP	197.0→92.0	-41	197.0→120.0	-32	M1
1-H-BTR	120.1→65.1	30	120.1→92.2	20	M1
1-OH-BTR	136.0→64.1	30			M1
5-Cl-BTR	154.0→99.3	30			M1
4-Me-1-H-BTR	134.1→79.0	35	134.1→77.1	35	M1
5-Me-1-H-BTR	134.1→79.0	35	134.1→77.1	35	M1
5,6-2Me-1-HBTR	148.0→93.3	30			M1
BTH	136.0→109.2	22	136.0→65.2	22	M1
2-OH-BTH	152.1→124.1	30	152.1→92.1	30	M1
2-Cl-BTH	170.1→93.0	39	170.1→65.0	53	M1
2-Me-BTH	150.0→108.8	31			M1
2-Me-S-BTH	182.0→109.1	50	182.0→65.1	70	M1
2-Mo-BTH	253.2→86.2	25	253.2→55.8	31	M1
2NH_2-BTH	151.0→124.1	22	151.0→109.1	22	M1
EHS	249.2→137.0	-24	249.2→93.0	-66	M1
HMS	249.2→137.0	-24	249.2→93.0	-66	M1
IAMC	249.2→161.0	20	249.2→76.7	70	M1
4-MBC	255.1→77.1	70	255.1→54.6	40	M1
ODPABA	278.2→151.0	40	278.2→166.0	30	M1
OMC	291.2→160.9	23	291.2→179.5	14	M1
UV-P	226.0→120.2	27	226.0→77.2	51	M1
UV-PS	268.2→212.2	23	268.2→65.2	73	M1
UV-234	448.1→91.0	83	448.1→370.3	30	M1

UV-320	324.2→57.0	40	324.2→212.1	40	M1
UV-326	316.1→260.2	30	316.1→107.2	40	M1
UV-327	358.1→301.9	31	358.1→246.0	35	M1
UV-328	352.1→282.2	25	352.1→70.9	27	M1
UV-329	322.1→289.0	-18	322.1→77.0	-22	M1
UV-350	324.2→268.2	23	324.2→212.1	40	M1
UV-360	659.3→238.0	31	659.3→207.0	35	M1

[a] **Details of instrumental methods:**

M1: The instrumental system consists of ultra-performance liquid chromatography coupled to a triple quadrupole mass spectrometer (Triple Quad 7500, AB Sciex, Toronto, Canada). Chromatographic separation of target analytes is achieved with a Luna® Omega 3 µm PS C18 (2) 100Å column (100 mm × 3.0 mm; 3 µm particle size; Phenomenex, Torrance, CA, U.S.). The mobile phase consists of water spiked with 0.2 mM ammonium acetate (solvent A) and methanol (solvent B), running at a flow rate of 400 µL/min. The following gradient is employed: 10% B (held for 2 min) ramped to 70% B over 4 min (linear), followed by a linear increase to 99% B over 11 min (held for 5 min) and then a linear change to 10% B (equilibrated for 3 min). The MS is equipped with a TurboIonSpray® electrospray ionization (ESI) probe and operated in multiple reaction monitoring (MRM) mode (positive and negative). The ESI source conditions are as follows: ion source gas 1, 35; ion source gas 2, 70; curtain gas (CUR), 40; collision gas (CAD), 9; source temperature, 320 °C; IonSpray voltage floating, 2000 V.

M2: The instrumental system consists of ultra-performance liquid chromatography coupled to a triple quadrupole mass spectrometer (Triple Quad 7500, AB Sciex, Toronto, Canada). Chromatographic separation of target analytes is achieved with a Luna® Omega 3 µm PS C18 (2) 100Å column (100 mm × 3.0 mm; 3 µm particle size; Phenomenex, Torrance, CA, U.S.). The mobile phase consists of water spiked with 0.2 mM ammonium acetate (solvent A) and methanol (solvent B), running at a flow rate of 250 µL/min. The following gradient is employed: 60% B (held for 1.5 min), ramped to 70% B over 7 min (linear), followed by a linear increase to 100% B over 1 min (held for 4 min) and then a linear change to 60% B (equilibrated for 3 min). The MS is equipped with a TurboIonSpray® electrospray ionization (ESI) probe and operated in negative multiple reaction monitoring (MRM) mode. The ESI source conditions are as follows: ion source gas 1, 35; ion source gas 2, 70; curtain gas (CUR), 40; collision gas (CAD), 9; source temperature, 320 °C; IonSpray voltage floating, 2000 V.

Table S5. Detection frequency (DF, %) and median concentrations (ng/mL) of exogenous chemicals in the urine of pregnant women in three cities. [a] No fill indicates no detectable blank contamination. [b] ND: not detected, with concentrations lower than limit of quantification.

			Shi Jiazhuang		Shang Hai		Cheng Du	
	LOQ (ng/mL)	Blank [a] (ng/mL, n=3)	median	DF	Median	DF	Median	DF
Anti-oxidants:								
**BHA**	0.0625		6.04	87%	2.44	84%	<LOQ	48%
**BHT**	0.0667		3.63	73%	4.78	97%	2.43	57%
**BHT-CHO**	0.0418	0.03	7.71	93%	3.21	90%	30.9	100%
**BHT-COOH**	0.0736		0.932	67%	4.11	99%	<LOQ	39%
**BHT-OH**	0.0333	0.05	0.185	75%	0.345	98%	<LOQ	16%
**BHT-Q**	0.0694	0.06	4.20	69%	2.45	71%	3.69	58%
**4-tOP**	0.0917	0.08	3.28	69%	2.20	85%	7.49	79%
**Cyanox 2246**	0.0036		0.130	67%	0.037	82%	0.385	96%
**DPG**	0.0385	0.03	0.253	72%	<LOQ	46%	2.46	84%
**Ethanox 703**	0.0714		<LOQ	36%	<LOQ	31%	<LOQ	18%
$\sum$**Synthetic antioxidants**			26.4		19.6		48.0	

Parabens:								
**Bzp**	0.0077		<LOQ	49%	0.032	85%	<LOQ	6%
**Etp**	0.0047		0.173	91%	0.425	94%	1.01	91%
**Mep**	0.0062		6.45	85%	49.7	95%	6.55	99%
**BuP**	0.0035		<LOQ	15%	0.033	58%	<LOQ	4%
**PrP**	0.0042		0.287	77%	1.26	98%	0.679	90%
**TCC**	0.0064		0.013	59%	0.659	99%	<LOQ	23%
**TCS**	0.0211		0.563	60%	1.22	70%	0.653	62%
∑**Personal care products**			7.49		53.3		8.89	
UV Stabilizers:								
**4-OH-BP**	0.0049		0.146	98%	0.157	98%	<LOQ	7%
**BP3**	0.0909		<LOQ	33%	1.01	73%	<LOQ	4%
**BP1**	0.0028		0.070	82%	0.418	89%	0.0519	51%
**BP8**	0.0127	0.04	0.087	68%	0.054	69%	<LOQ	16%
**5,6-2Me-1-HBTR**	0.0278		<LOQ	4%	<LOQ	9%	<LOQ	9%
**1-H-BTR**	0.0138		1.31	81%	1.78	71%	1.30	52%
**4/5-Me-1-H-BTR**	0.0103		<LOQ	1%	<LOQ	18%	<LOQ	20%
**OC**	0.0167		0.240	64%	<LOQ	46%	<LOQ	36%

**4-MBC**	0.0282		<LOQ	6%	<LOQ	23%	<LOQ	6%
**OMC**	0.0077		1.64	93%	0.845	73%	1.26	87%
**BMDM**	0.0333		<LOQ	2%	<LOQ	33%	<LOQ	13%
$\sum_{\text{UV stabilizers}}$			9.46		11.4		1.31	
OPFRs:								
**TBEP**	0.0370		0.049	54%	0.473	89%	<LOQ	14%
**TBP**	0.0041		0.026	53%	0.047	68%	0.0135	55%
**TCPP**	0.0690		0.694	60%	<LOQ	18%	<LOQ	4%
**TEP**	0.0062		4.65	62%	0.445	53%	<LOQ	26%
**TEHP**	0.0625	0.04	<LOQ	18%	<LOQ	21%	0.127	51%
**TPHP**	0.0079		0.096	67%	<LOQ	34%	<LOQ	36%
**BBOEP**	0.0476		0.930	80%	3.06	93%	1.49	91%
**BEHP**	0.0909		<LOQ	25%	<LOQ	36%	0.109	51%
**DBP**	0.0649	0.05	2.35	96%	0.315	62%	<LOQ	40%
**DEP**	0.0719		3.45	98%	6.46	90%	21.8	100%
**DMP**	0.0775	0.05	3.06	99%	3.46	99%	<LOQ	49%
$\sum_{\text{Organophosphate esters}}$			15.3		14.3		77.7	
Mono-phthalates:								

**mCMHP**	0.250		3.29	96%	2.70	100%	5.11	97%
**mECPP**	0.286		3.12	96%	2.37	99%	5.58	100%
**mEHHP**	0.125		16.2	100%	10.3	100%	13.8	100%
**mEOHP**	0.0909		2.89	100%	1.69	100%	3.71	100%
**mHiNP**	0.0909		3.86	99%	2.61	100%	7.07	100%
**miBP/mBP**	0.0600	0.04	39.9	100%	22.3	100%	56.5	100%
**mEHP/ [illegible]**	0.118		3.73	100%	4.07	100%	0.816	80%
**mBzP**	0.0840		0.310	84%	0.724	93%	<LOQ	28%
**mCHP**	0.0559		1.17	90%	0.094	52%	1.80	92%
**mETP**	0.313		6.45	99%	4.88	100%	2.90	95%
**miNoP**	0.111		4.44	93%	4.94	90%	<LOQ	6%
**mMp**	0.575	0.31	299	100%	566	99%	372	100%
**∑Mono-phthalate esters**			372		618		470	
Plasticizers:								
**TCaT/TCTM**	0.0102		0.269	59%	0.098	53%	1.24	59%
**DiBA/DnBA**	0.0625	0.05	7.44	96%	7.06	87%	38.2	100%
**TBC**	0.0193		4.06	97%	1.64	88%	0.853	75%
**DINCH**	0.0667		<LOQ	28%	<LOQ	31%	<LOQ	39%

**ATBC**	0.0649		8.58	86%	1.70	63%	16.0	97%
**DEHM**	0.0080		<LOQ	13%	0.476	71%	0.536	88%
**DBF**	0.0323		0.130	52%	<LOQ	37%	<LOQ	44%
$\sum_{\textbf{non-PAE plasticizers}}$			20.2		11.0		56.8	
Photoinitiator additives:								
**BP**	0.0189		1.85	83%	<LOQ	42%	0.0935	52%
**MK**	0.0015		0.031	85%	0.015	71%	<LOQ	50%
$\sum_{\textbf{Photoinitiator additives}}$			1.88		0.0150		0.100	
Pesticides:								
**DUB**	0.0345		<LOQ	7%	<LOQ	18%	0.0838	53%
**THI**	0.0074		<LOQ	13%	<LOQ	8%	<LOQ	ND[b]
**THX**	0.0909		1.52	89%	<LOQ	16%	0.537	57%
**DCV**	0.0313	0.02	2.17	96%	1.19	88%	<LOQ	28%
**DCP**	0.0625		3.43	99%	3.74	100%	<LOQ	33%
**3-PBA**	0.0193		0.759	93%	1.08	100%	2.07	93%
**PNP**	0.0200		0.561	62%	2.36	88%	3.45	73%
**3Me4NP**	0.0103		0.052	51%	0.105	78%	3.14	72%
**4F3PBA**	0.0714		0.863	96%	0.803	99%	1.86	65%

∑Pesticides			9.36		9.28		11.1	
OH-PAHs:								
**2-OH-Nap**	0.0166	0.01	1.85	94%	3.96	97%	15.5	100%
**1-OH-Nap**	0.0143		<LOQ	42%	0.917	80%	11.1	96%
**2-OH-Flu**	0.0076		0.620	93%	1.77	95%	6.55	100%
**3-OH-Flu**	0.0076		<LOQ	40%	0.073	59%	0.422	96%
**9-OH-Flu**	0.0076		<LOQ	39%	0.104	60%	0.146	64%
**2-OH-Phe**	0.0265		0.446	98%	1.11	100%	2.36	100%
**3-OH-Phe**	0.0265		0.482	99%	1.08	100%	3.76	100%
**1/9-OH-Phe**	0.0265		0.707	96%	1.45	99%	2.59	100%
**4-OH-Phe**	0.0265		0.235	86%	0.327	96%	0.103	54%
**1-OH-Pyr**	0.0063		0.198	99%	0.256	99%	0.183	95%
**3-OH-BcP/ [illegible]**	0.0016		<LOQ	32%	0.008	65%	<LOQ	1%
∑OH-PAHs			4.54		11.1		42.7	

Table S6. Results of one-way ANOVA analysis.

	Square Sum (SS)	Degrees of Freedom (df)	Mean Square (MS)	F	p-Value
Between	0.814	2	0.407	5.550	0.004
Within	35.480	484	0.073		
Total	336.294	486			

Table S7. Association between concentrations of highly detected chemicals in the urine of pregnant women and the risk of gestational diabetes mellitus. All models were adjusted for maternal age (years), pre-pregnancy BMI (kg/m^2), income (CNY/year), maternal education, parental history of diabetes, smoking, and alcohol drinking. [a] OR: odds ratio.

	**OR[a]**	**5%**	**95%**	**p-value (FDR)**
**3-PBA**	0.895	0.757	1.057	0.408
**3Me4NP**	1.024	0.927	1.133	0.749
**PNP**	1.020	0.914	1.141	0.820
**mMP**	0.960	0.686	1.349	0.836
**mETP**	0.927	0.728	1.180	0.643
**mCHP**	0.993	0.813	1.219	0.946
**mEHP/mOcP**	0.904	0.721	1.131	0.529
**miBP/mBP**	0.726	0.548	0.954	0.107
**mHiNP**	0.861	0.666	1.107	0.481
**mEOHP**	0.796	0.601	1.045	0.297
**mEHHP**	0.819	0.624	1.067	0.350
**mECPP**	0.772	0.587	1.006	0.197
**mCMHP**	0.755	0.590	0.959	0.107
**DEP**	0.621	0.429	0.887	0.065
**BBOEP**	0.928	0.778	1.106	0.529
**EtP**	0.986	0.883	1.102	0.836
**MeP**	1.074	0.914	1.266	0.529
**ProP**	1.048	0.939	1.173	0.529
**DPG**	1.162	1.005	1.354	0.176
**Cyanox2246**	1.081	0.897	1.307	0.529
**4-tOP**	1.217	1.053	1.422	0.065
**BHT-CHO**	1.193	0.832	1.725	0.529
**OMC**	0.943	0.783	1.121	0.640
**ATBC**	1.419	1.167	1.752	**0.025**
**DEHM**	1.081	0.918	1.282	0.529
**DiBA/DnBA**	1.225	0.905	1.688	0.408
**TBC**	1.097	0.958	1.262	0.408
**2-OH-Nap**	0.968	0.781	1.199	0.830
**1-OH-Nap**	0.831	0.716	0.955	0.065

**1-OH-Pyr**	0.918	0.763	1.105	0.529
**1/9-OH-Phe**	0.781	0.564	1.071	0.340
**3-OH-Phe**	0.604	0.420	0.856	0.065
**2-OH-Phe**	0.691	0.483	0.977	0.160
**3-OH-Flu**	0.918	0.772	1.096	0.529
**2-OH-Flu**	0.626	0.440	0.878	0.065

Table S8. Posterior inclusion probabilities (PIPs) from BKMR.

Chemicals	PIPs
2-OH-Nap	0.651
1-OH-Nap	0.480
2-OH-Flu	0.446
3-OH-Flu	0.459
2-OH-Phe	0.492
3-OH-Phe	0.498
1/9-OH-Phe	0.398
1-OH-Pyr	0.297
TBC	0.341
DiBA/DnBA	0.453
DEHM	0.356
ATBC	0.940
OMC	0.396
BBOEP	0.380
DEP	0.435
BHT/CHO	0.274
4-tOP	0.889
Cyanox2246	0.376
DPG	0.272
ProP	0.896
MeP	0.262
EtP	0.125
mCMHP	0.569
mECPP	0.376
mEHHP	0.406
mEOHP	0.379
mHiNP	0.419
miBP/mBP	0.442
mEHP/mOcP	0.302
mCHP	0.369
mETP	0.277
mMP	0.356
PNP	0.193
3Me4NP	0.153
3-PBA	0.329

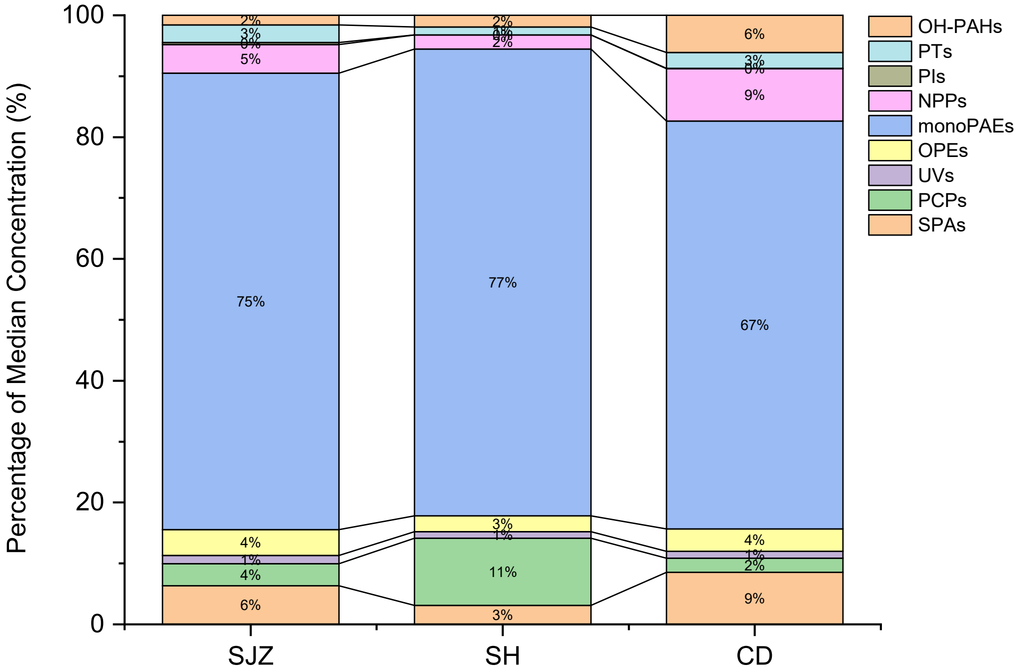

Figure S1. Composition profiles of major compounds in the urine of pregnant women in three cities.

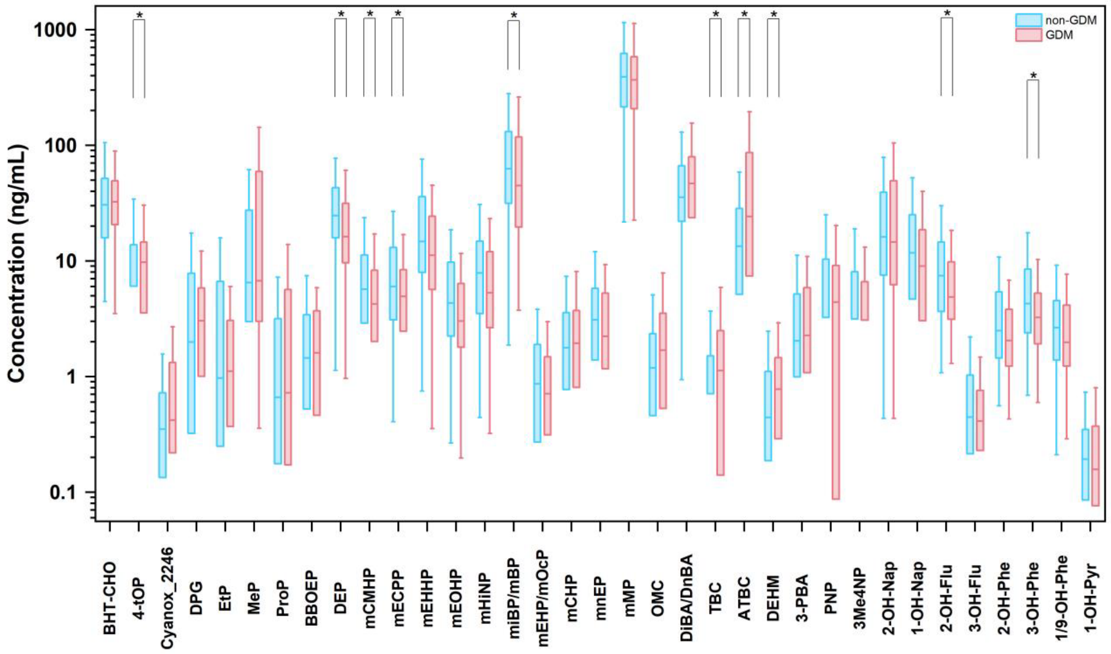

Figure S2. Differences in the concentration of chemicals (DF>70%) in the urine of pregnant women in the case and control groups. The top and bottom edges of the box represent the third and first quartiles of the corresponding chemicals, respectively. The horizontal lines in the box represent the median concentration of the chemicals. The top and bottom whiskers represent the 5% and 95% quartile concentrations of the chemicals, respectively. *: P<0.05.

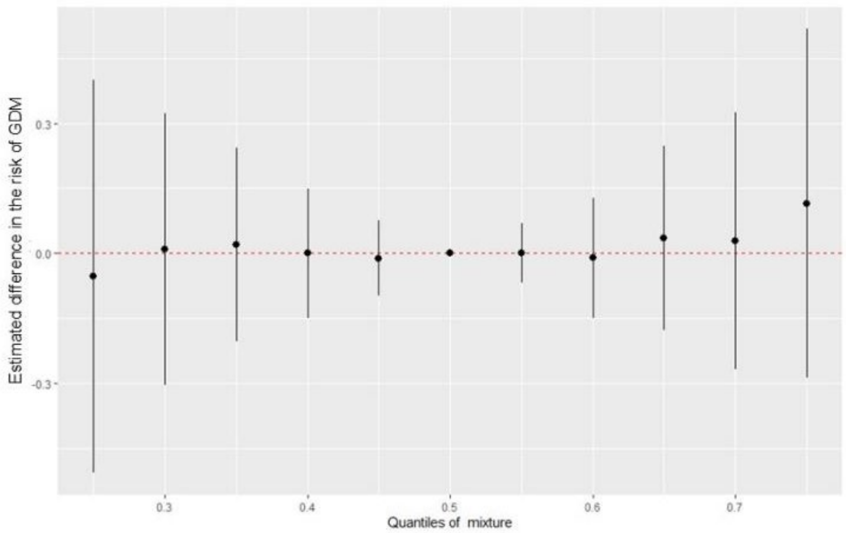

(**a**)

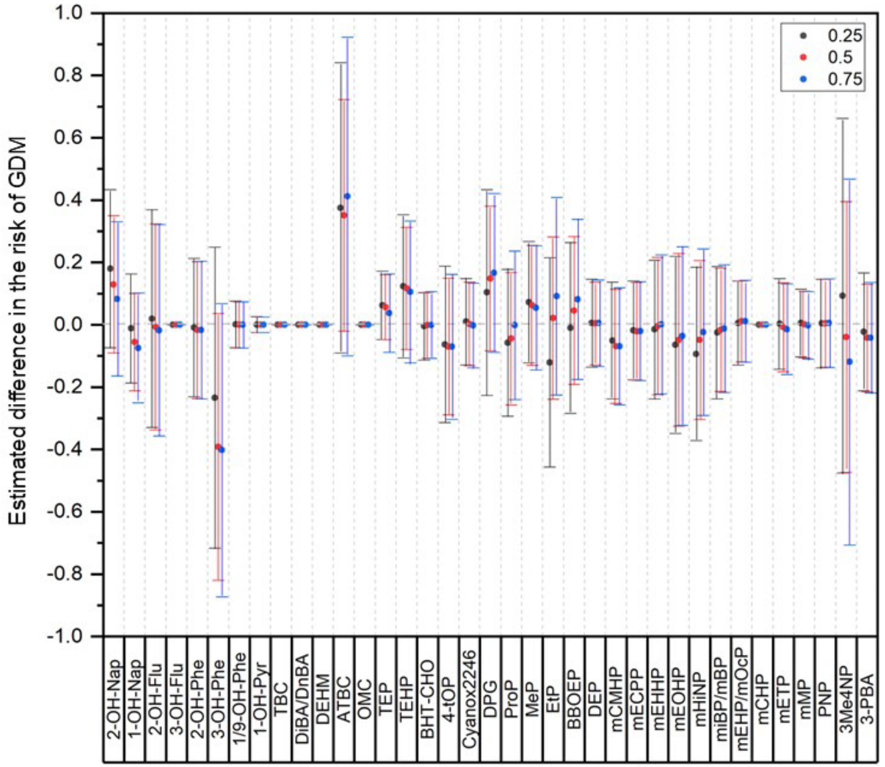

(**b**)

Figure S3. Associations of the chemicals mixture with GDM risk. Models were adjusted for maternal age, pre-pregnancy BMI, maternal education, alcohol drinking, smoking during pregnancy, and parental history of diabetes. (a) Overall association between chemical mixtures (estimates and 95% CIs) and GDM risk. The figure plots the estimated difference in GDM risk when exposures are at a particular percentile (x-axis) in comparison with when exposures are all at the 50th percentile. (b) Single-chemical association (estimates and 95% CIs). The plot compares the risk of GDM when a single chemical is at the 75th vs 25th percentile, while all the other chemicals are fixed at either the 25th, 50th, or 75th percentile.
